# Occupational exposure to radiofrequency electromagnetic fields and brain tumor risk: Application of the INTEROCC job‐exposure matrix

**DOI:** 10.1002/ijc.35182

**Published:** 2024-09-20

**Authors:** Maxime Turuban, Hans Kromhout, Javier Vila, Miquel Vallbona‐Vistós, Frank De Vocht, Isabelle Baldi, Lesley Richardson, Geza Benke, Daniel Krewski, Marie‐Elise Parent, Siegal Sadetzki, Brigitte Schlehofer, Joachim Schüz, Jack Siemiatycki, Martie van Tongeren, Alistair Woodward, Elisabeth Cardis, Michelle C. Turner

**Affiliations:** ^1^ Barcelona Institute for Global Health (ISGlobal) Barcelona Spain; ^2^ Universitat Pompeu Fabra (UPF) Barcelona Spain; ^3^ Institute for Risk Assessment Sciences (IRAS) Utrecht University Utrecht The Netherlands; ^4^ Environmental Protection Agency (EPA) Office of Radiation Protection and Environmental Monitoring Wexford Ireland; ^5^ Population Health Sciences, Bristol Medical School University of Bristol Bristol UK; ^6^ NIHR Applied Research Collaboration West (NIHR ARC West) Bristol UK; ^7^ INSERM UMR 1219 Epicene Team Bordeaux Population Health Research Center Bordeaux France; ^8^ Service Santé Travail Environnement CHU de Bordeaux Bordeaux France; ^9^ University of Montreal Hospital Research Centre (CRCHUM) Montreal Canada; ^10^ School of Public Health and Preventive Medicine Monash University Melbourne Australia; ^11^ School of Epidemiology and Public Health University of Ottawa Ottawa Ontario Canada; ^12^ Institut National de la Recherche Scientifique Université du Québec Laval Quebec Canada; ^13^ Faculty of Medicine Tel‐Aviv University Tel‐Aviv Israel; ^14^ Private Leimen Germany; ^15^ International Agency for Research on Cancer (IARC) Environment and Lifestyle Epidemiology Branch Lyon France; ^16^ Division of Population Health, Health Services Research and Primary care University of Manchester Manchester UK; ^17^ School of Population Health University of Auckland Auckland New Zealand; ^18^ Spanish Consortium for Research and Public Health (CIBERESP) Instituto de Salud Carlos III Madrid Spain

**Keywords:** brain tumors, INTEROCC, job‐exposure matrix, occupational exposure, radiofrequency electromagnetic fields

## Abstract

Radiofrequency electromagnetic fields (RF‐EMF, 100 kHz to 300 GHz) are classified by IARC as possibly carcinogenic to humans (Group 2B). This study evaluates the potential association between occupational RF‐EMF exposure and brain tumor risk, utilizing for the first time, a RF‐EMF job‐exposure matrix (RF‐JEM) developed in the multi‐country INTEROCC case–control study. Cumulative and time‐weighted average (TWA) occupational RF‐EMF exposures were estimated for study participants based on lifetime job histories linked to the RF‐JEM using three different methods: (1) by considering RF‐EMF intensity among all exposed jobs, (2) by considering RF‐EMF intensity among jobs with an exposure prevalence ≥ the median exposure prevalence of all exposed jobs, and (3) by considering RF‐EMF intensity of jobs of participants who reported RF‐EMF source use. Stratified conditional logistic regression models were used, considering various lag periods and exposure time windows defined a priori. Generally, no clear associations were found for glioma or meningioma risk. However, some statistically significant positive associations were observed including in the highest exposure categories for glioma for cumulative and TWA exposure in the 1‐ to 4‐year time window for electric fields (E) in the first JEM application method (odds ratios [ORs] = 1.36, 95% confidence interval [95% CI] 1.08, 1.72 and 1.27, 95% CI 1.01, 1.59, respectively), as well as for meningioma for cumulative exposure in the 5‐ to 9‐year time window for electric fields (E) in the third JEM application method (OR = 2.30, 95% CI 1.11, 4.78). We did not identify convincing associations between occupational RF‐EMF exposure and risk of glioma or meningioma.

## What's New?

Radiofrequency electromagnetic fields have been classified as possibly carcinogenic to humans. Here, the authors applied a novel job‐exposure matrix to investigate the link between occupational radiofrequency electromagnetic field exposure and brain tumors within the multi‐country INTEROCC case–control study. The findings revealed no clear associations, despite some positive findings for gliomas and meningiomas in the 1‐ to 4‐ and 5‐ to 9‐year exposure windows. The study underlines the applicability of the job‐exposure matrix in occupational research and the need to further investigate the association between brain tumors and occupational radiofrequency electromagnetic field exposure.

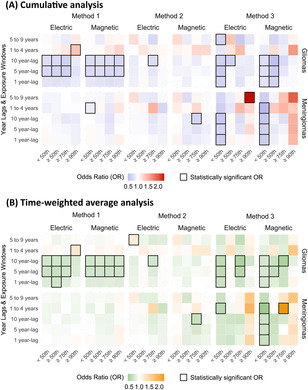

## 1 INTRODUCTION

Radiofrequency electromagnetic fields (RF‐EMF) and their potential health effects have been the subject of scientific investigation for decades.^1^ RF‐EMF, spanning frequencies from 100 kHz to 300 GHz, are used in diverse applications. They are commonly used to transmit information wirelessly for broadcasting, telecommunication, and RF identification; they function in remote sensing tools such as radars and security scanners. They are also employed for the heating and/or drying of products, as well as in some medical diagnostic and therapeutic procedures.^2^, ^3^ At high intensity, RF‐EMF have the potential to generate excessive heat inside the body, which may cause tissue damage. To mitigate health risks, guidelines have been established by agencies such as the International Commission on Non‐Ionizing Radiation Protection (ICNIRP).^4^ While the general public encounters relatively low intensity levels of these RF fields emitted by everyday technologies, workers in certain professions might experience higher and more sustained RF‐EMF exposure due to their proximity to, or use of, specific devices or sources emitting RF‐EMF in occupational settings. Worker exposure levels to RF‐EMF have been described in various sectors including in healthcare,^5^, ^6^, ^7^ telecommunications,^8^, ^9^, ^10^ manufacturing,^11^, ^12^, ^13^ armed forces^14^, ^15^ as well as among office workers.^16^, ^17^ Occupational standards were occasionally exceeded in many of these sectors,^18^ but exposure for office workers was considered equivalent to that of the general population.^17^

In 2011, a Working Group convened by the International Agency for Research on Cancer (IARC) classified RF‐EMF as Group 2B, possibly carcinogenic to humans, with some evidence of an increased risk of glioma and acoustic neuroma.^1^ Recently, an Advisory Group to the IARC Monographs recommended the re‐evaluation of the carcinogenicity of RF‐EMF with high priority based on new evidence from human and animal studies.^19^ While many studies have explored the potential association between RF‐EMF and cancer, primarily in relation to mobile phone use, fewer studies have investigated this association in occupational settings.^15^, ^20^, ^21^ Moreover, occupational studies have typically been limited by the methods used to ascertain worker exposure. To further address these questions, better methods of occupational exposure assessment that can be applied in large‐scale epidemiological studies are needed.

In previous occupational epidemiological studies, exposure assessment often relied on surrogates of exposure such as job titles, work in proximity to a particular RF‐EMF source, or distance to the source.^22^, ^23^ Qualitative exposure estimates were commonly assigned by occupational hygienists or derived from JEMs based on expert judgment. The few studies that employed exposure estimates derived from measurement data, typically involved a small number of measurements and did not account for temporal variation in exposure levels.^24^, ^25^

A prior epidemiological analysis utilizing a source‐exposure matrix (SEM) approach in the INTEROCC study did not reveal any notable associations between cumulative occupational RF‐EMF exposure and either glioma or meningioma risk.^21^ The SEM was created by integrating exposure data, primarily spot‐measurements, extracted from the literature and summarized in an Occupational Exposure Measurement Database (OEMD), with occupational source‐based questionnaire information collected in INTEROCC.^2^, ^3^ Some nonsignificant positive odds ratios (ORs) were observed in the highest exposed groups for recent exposures (experienced in the 1–4 years preceding diagnosis/reference date) for both glioma and meningioma. The prevalence of exposure was low.^21^

The construction of the INTEROCC RF job‐exposure matrix (RF‐JEM) has been detailed elsewhere.^26^ In short, the RF‐JEM was constructed using exposure estimates compiled in the SEM (above) coupled with occupational information captured by INTEROCC questionnaires per job. Information included the types of sources used, their frequency, and the number of INTEROCC participants in each job code who reported using a particular relevant source. By combining these data, exposure intensity and prevalence metrics for electric (E) and magnetic (H) fields were estimated across 468 four‐digit ISCO88 occupations.^26^ Given that RF electric and magnetic fields are only proportional in the far‐field—typically beyond one wavelength from the source, separate assessments of electric (E) and magnetic (H) fields are essential. They behave independently in near‐field conditions, where proximity to the source alters their interaction with the body.^27^ A JEM approach including exposure estimates for both E and H fields provides standardized, job‐level exposure estimates for broad use in epidemiological research.^28^ The database of jobs held by INTEROCC subjects had already been previously linked to other JEMs such as the INTEROCC chemical JEM^29^ to study the relationship between brain tumors and a range of chemical exposures, as well as with extremely low frequency (ELF) EMF through an updated version of an ELF‐JEM.^30^

The objective of this article is to report results of the first analysis using the INTEROCC RF‐JEM within the INTEROCC study population to examine the potential association between occupational RF‐EMF exposure and risk of glioma and meningioma. The RF‐JEM was linked to occupational histories in three ways: (1) by considering RF intensity estimates among all exposed jobs, (2) by considering RF intensity estimates only among jobs with an exposure prevalence ≥the median exposure prevalence of all exposed jobs, and (3) by considering RF intensity estimates of jobs for participants who also reported RF‐EMF source use in detailed occupational questionnaire modules. We also compare our present findings based on the RF‐JEM with previous results based on the SEM in an attempt to evaluate the performance of the RF‐JEM.^21^

## 2 METHODOLOGY

### 2.1 Study population

The INTEROCC study population is based on seven of the 13 INTERPHONE countries that participated in this case–control study of risk factors for primary glioma and meningioma between 2000 and 2004^31^: Australia, Canada, France, Germany, Israel, New Zealand, and the United Kingdom. The age range of participants varied among countries. While the core INTERPHONE protocol targeted individuals aged 30–59 years, the inclusion criteria differed among countries: in Germany, subjects up to the age of 69 years were included; in the United Kingdom, the age range was 18–69 years; and in Israel, individuals aged 18 years and above were included without an upper age limit. Controls were randomly selected from the source population, mainly using population registries, electoral lists, general practitioner patient lists in the United Kingdom, and random digit dialing in Ottawa, Canada. To improve efficiency, controls were matched to cases by age group, sex, study region, and country, maintaining an approximate 1:1 case–control ratio, except in Germany, where 1:2 matching was used. Upon contact, potential participants were briefed on the study, and those agreeing to participate signed an informed consent prior to interview.^31^

The INTEROCC study subject database has been described elsewhere.^21^, ^29^, ^30^, ^32^, ^33^, ^34^, ^35^ Briefly, information was collected from 2054 cases of glioma, 1924 cases of meningioma, and 5601 controls. Participation rates were 69% and 79% for glioma and meningioma cases respectively, and 50% for controls.^36^ The main reasons people did not take part were refusal (64%) or being unreachable (27%). If a person had died or otherwise could not take part, a proxy was allowed to answer for them, proxy interviews were used for 13% of glioma cases.^31^

### 2.2 Data collection

Whenever possible, study subjects were interviewed in person by trained interviewers using a computer‐assisted personal interview. The study questionnaire included, in particular, information on demographic, lifestyle and socioeconomic factors, and occupational history, with a comprehensive job history including all jobs held for at least 6 months or longer. The information obtained from participating subjects included a lifetime list of job titles, start and end dates, company name and description, and detailed tasks. An occupational hygienist in each country coded the local jobs according to the International Standard Classification of Occupations from both the 1968 system (ISCO68), with three and five‐digit codes, and the 1988 system (ISCO88), with two, three, and four‐digit codes.

Exposure ascertainment covered electromagnetic fields ranging from 0 Hz to 300 GHz. As such, the questionnaire contained targeted screening questions to identify those who might have worked with or near EMF sources in particular settings where substantial exposure could occur, including (i) medical diagnosis and treatment, (ii) food‐heating equipment (for cooking, drying, sterilizing, or pasteurizing food), (iii) industrial heating, (iv) semiconductor manufacturing, (v) radars, (vi) telecommunication antennas, and (vii) portable transmitters such as CB radios or walkie‐talkies. This led to further inquiries about the nature of the work, the job(s) in which this took place, the exposure sources, tasks, work arrangements, start and end years, and number of hours per week/month the subject worked with or near the reported source(s).^2^ A subject could report multiple sources of exposure and, in some instances, multiple questionnaire modules for a given job were completed.

### 2.3 Exposure assessment

In 2019, a RF‐JEM was created with estimates of exposure to electric (E) and magnetic (H) fields based on the INTEROCC study SEM and subjects' coded occupational histories.^26^ The RF‐JEM was based on the international ISCO88 coding system, giving estimates of both intensity and prevalence of RF‐EMF exposure for each four‐digit ISCO88 code found in INTEROCC. The JEM provides exposure estimates for 468 ISCO88 four‐digit codes, of which 62% were considered as occupationally exposed to frequency ranges including intermediate‐frequency ELF (IF‐ELF; 3–100 kHz), intermediate‐frequency radiofrequency (IF‐RF; 100 kHz–10 MHz), or RF (10 MHz–300 GHz); with the majority of exposed job codes exposed to RF only (88% of exposed job codes for E‐fields and 87% for H fields).^26^

Intensity estimates in the JEM were computed by combining the mean electric (E) or magnetic (H) field strength extracted from the SEM estimates for the different sources encountered in each job code, depending on their frequency range, and the corresponding frequency‐specific ICNIRP basic restrictions (BRs) and reference levels (RLs). For IF‐RF and RF (>100 kHz), intensity was calculated as the E‐ and H‐field ICNIRP squared ratios (Equation 1). These ratios were used to consider the frequency‐dependent biophysical responses to EMF exposure, and the multiplicity of frequency ranges within the same band (i.e., RF frequencies in kHz, MHz, or GHz). ICNIRP's guidelines include frequency‐specific RLs for occupational exposure, based on thresholds of biological responses, or BRs.^4^ The fact that these guidelines vary with frequency emphasize the necessity of accommodating frequency variations in the assessment of exposure.

(1) $$\text{ICNIRP squared rati}o_{\mathrm{Gs}\left(f\right)}=\frac{{\left[\bar{G_{s}}\left(f\right)\right]}^{2}}{{\left[G_{\mathrm{RLs}}\left(f\right)\right]}^{2}}\propto \mathrm{SAR}$$

In Equation (1), G_*S*_(*f*) denotes the electric (E) field in V/m or the magnetic (H) field in A/m for a source *S* with frequency *f*. $\bar{G_{s}}$ (*f*) is the arithmetic mean E or H exposure level for that source and frequency, taken from the SEM. $G_{\mathrm{RLs}}$(*f*) is the ICNIRP standard RL for the frequency of that source. For frequencies above 100 kHz, ICNIRP squared ratios are thought to approximate the specific absorption rate (SAR).^26^ Estimates for each source were then combined within their respective job codes along additional modifying factors (i.e., operating distance from source, proportion of time exposed to the source during a shift) to obtain a single time‐weighted average (TWA) ICNIRP squared ratio estimate for each exposed ISCO88 job code. Since INTEROCC focused on brain tumor risk, measurements in the SEM made at the head level were also upweighted during an expert confidence‐rating assessment. Further details on the RF‐JEM creation are available elsewhere.^26^

We linked the RF‐JEM to the occupational histories of cases and controls using three increasingly restrictive methods for exposure assessment, in order to gain insight into the performance of the JEM. (1) We applied RF intensity scores to all exposed job titles, considering participants with a job history without RF‐exposed jobs as nonexposed (Method 1). (2) In order to have fewer false positives, we created a more specific version of the JEM by only assigning exposure estimates for jobs with RF exposure prevalences above the median prevalence of exposure among all exposed jobs (5.1% for E fields, 4.8% for H fields, and 1.5% for both E and H fields in IF‐RF). Occupations with an exposure prevalence below the median prevalence were considered nonexposed (Method 2). (3) Finally, we assigned exposure estimates from the RF‐JEM exclusively to those jobs for which individual participants reported RF‐EMF source use in detailed occupational modules ascertained through the study questionnaire (Method 3). We also considered various exposure lags and time windows in the analysis.^21^

### 2.4 Statistical analysis

Analyses were performed across the full RF‐EMF frequency spectrum, therefore including both IF‐RF and RF‐EMF frequencies and summing them at the job level. In order to calculate lifetime‐ or time window‐specific cumulative IF‐RF/RF‐EMF (100 kHz–300 GHz) electric (E) and magnetic (H) field exposure estimates using JEM information, the number of years a participant worked in a specific ISCO88 code was multiplied by the relevant intensity level estimate from the JEM. This estimate was then summed across all occupations held by the participant up to the diagnosis/reference date minus a lag period. We considered multiple lag periods (1, 5, and 10 years) as well as two specific exposure time windows (1–4 and 5–9 years) before the diagnosis/reference date. TWA exposure estimates were also calculated by dividing the cumulative exposure estimate by the number of years worked in a given job, for each lag period and specific exposure time window.

Participants who reported jobs with unknown start or end dates (in years) or those containing incoherent chronological information were excluded from the analysis. If a participant had overlapping jobs, the exposure estimates were averaged for those overlapping years. Participants who presented uncertain information regarding the use or exposure to any source were similarly excluded. The exclusion criteria were applied to maintain comparability across the different RF‐JEM application approaches. Participants who exclusively reported nonstandard occupations (housewife/husband, pensioners, training, or imprisonment) in their occupational history were excluded. If these nonstandard occupations were reported alongside other jobs, they were retained in the analysis and considered as nonexposed periods. All participants with any unknown occupation were excluded.

Associations between exposure to IF‐RF/RF‐EMF (with E‐fields and H‐fields considered separately), and the risk of glioma or meningioma were investigated using stratified conditional logistic regression models. These models, which estimated ORs and 95% confidence intervals (CIs), were stratified according to the original matching factors (age in 5‐year intervals, sex, and country/region) and adjusted for education. All eligible controls were used in the analysis to maximize the available sample size. The analyses were conducted with specific exposure lags and time windows of exposure selected a priori as in previous work, to explore if more recent or distant exposures to E or H fields of IF‐RF/RF‐EMF could be associated with varying brain tumor risk including possibly related to the promotion or progression of tumors in the most recent exposure time periods.^21^ For cases, the date of diagnosis was the reference date, whereas for controls, it was the interview date less the median time span between diagnosis and interview dates among cases.

Exposure to occupational IF‐RF/RF‐EMF was examined using categorical indicators of E and H fields. Given the skewed nature of the exposure data, cut‐points were used to define distinct categories across the spectrum of exposure (50th, 75th, and 90th percentiles).^37^ These cut‐points were calculated based on the exposure distribution among controls. Participants who were never exposed to occupational IF‐RF/RF‐EMF served as the reference group for the primary analysis. We aggregated both IF‐RF and RF exposures together for each ISCO88 code as individuals in these occupations could potentially be exposed to both types of frequencies and because ICNIRP squared ratios are used for both frequencies (>100 kHz).

Sensitivity analyses were performed to assess potential bias. We conducted a targeted assessment to address potential biases in glioma cases regarding the final years worked. Specifically, we limited our analysis to cases and controls who were actively employed throughout the entire most recent 1‐ to 4‐year time period before the diagnosis/reference date to mitigate the potential impact of reduced work years in glioma cases due to the onset of symptoms of disease. We examined exposure associations separately for low‐grade (I–II) and high‐grade (III–IV) glioma cases. Additionally, we assessed exposure using additional year lags (15, 20, and 25 years) and specific time windows of exposure (10–14, 15–19, and 20–24 years) to evaluate longer latency effects. As in previous analyses with the INTEROCC dataset using the SEM,^21^ using the low‐exposed group as the reference category instead of the nonexposed group. Sensitivity analyses also excluded participants with proxy responses, those over 60 years of age at time of interview, individuals with the highest exposure levels (>99th percentile) and those with specific pre‐existing medical conditions such as neurofibromatosis or tuberous sclerosis. Finally, analyses were also carried out by sex.^21^ Statistical analysis was performed with R software version 4.2.2.^38^ Conditional logistic regression analysis were performed using the “survival” package.^39^

## 3 RESULTS

In total, 260 controls (4.6%), 137 glioma cases (6.7%), and 115 meningioma cases (6.0%) were excluded due to incomplete or unknown RF‐EMF source information and lack of occupational history information. Participants excluded due to missing education level represented 11 controls (0.2%), 14 glioma cases (0.7%), and 7 meningioma cases (0.4%). Chronological issues in occupational history led to the exclusion of 29 controls (0.5%), 47 glioma cases (2.3%), and 15 meningioma cases (0.8%). Finally, 32 controls (0.6%), 12 glioma cases (0.6%), and 20 meningioma cases (1.0%) were excluded for having worked only in nonstandard occupations and for having any unknown occupation in their job history (42 controls, 0.7%; 25 glioma cases, 1.2%; and 9 meningioma cases, 0.5%). This resulted in a total of 1819 glioma cases, 1758 meningioma cases, and 5227 controls included for the analysis.

Sociodemographic characteristics of included participants are given in Table 1. Among glioma cases, the highest percentages were observed in the 50–54 (17.8%) and 55–59 year (15.7%) age groups. Males constituted 60.7% of glioma cases, but only 44.2% of controls. The largest numbers of glioma cases came from the United Kingdom (29.5% of the total), followed by Israel (20.7%), and Germany (19.0%). Regarding education, 52.4% of glioma cases had high school or lower education, compared to 53.9% of controls. For meningioma cases, 73.2% were female. Their age distribution was similar to glioma showing the highest frequencies in the 50–54 (20.1%) and 55–59 (17.2%) year age groups. Israel contributed the highest percentage of meningioma cases (36.8%). In terms of education, 59.2% had at most a high school education. Both glioma and meningioma cases had comparable mean numbers of years worked (up to 1 year before the diagnosis date), with averages of 27.6 ± 12.1 years for glioma cases, 27.4 ± 12.2 for meningioma cases, and 27.6 ± 11.6 for controls.

**TABLE 1:** Sociodemographic characteristics of included participants by age, sex, country, and education: INTEROCC study (2000–2004).

	Glioma (*n* = 1819)		Meningioma (*n* = 1758)		Controls (*n* = 5227)	
Variables	*n*	%	*n*	%	%	%
Age groups (years)						
<35	200	11.0	76	4.3	401	7.7
35–39	169	9.3	96	5.5	443	8.5
40–44	204	11.2	163	9.3	604	11.6
45–49	224	12.3	261	14.8	713	13.6
50–54	324	17.8	354	20.1	930	17.8
55–59	286	15.7	303	17.2	972	18.6
60–64	180	9.9	174	9.9	488	9.3
65–69	127	7.0	156	8.9	416	8.0
70+	105	5.8	175	10.0	260	5.0
Sex						
Female	715	39.3	1287	73.2	2917	55.8
Male	1104	60.7	471	26.8	2310	44.2
Country						
Australia	257	14.1	239	13.6	633	12.1
Canada	154	8.5	90	5.1	613	11.7
France	89	4.9	138	7.8	456	8.7
Germany	346	19.0	364	20.7	1478	28.3
Israel	377	20.7	647	36.8	898	17.2
New Zealand	60	3.3	48	2.7	136	2.6
United Kingdom	536	29.5	232	13.2	1013	19.4
Education						
High school or less	954	52.4	1040	59.2	2818	53.9
Medium‐level technical school	351	19.3	344	19.6	978	18.7
University	514	28.3	374	21.3	1431	27.4

A summary of the distribution of cumulative and TWA exposures to IF‐RF/RF‐EMF for glioma cases and controls with different lag‐times and for specific time windows is presented in Table 2A (E fields) and Table 2B (H fields). Equivalent tables for meningioma are given in Tables S1a and S1b.

**TABLE 2A:** Cumulative and average estimates of exposure based on three methods of attributing RF‐JEM estimates for glioma cases (*n* = 1819) and controls (*n* = 5227).

Exposure period	Participants status	Exposure assessment Method 1					Exposure assessment Method 2					Exposure assessment Method 3				
		%^a^	Mean (SD)^b^	50th^c^	75th^c^	90th^c^	%^a^	Mean (SD)^b^	50th^c^	75th^c^	90th^c^	%^a^	Mean (SD)^b^	50th^c^	75th^c^	90th^c^
Cumulative exposure																
1‐year lag	Controls	94.5	35.1 (64.3)	17.9	40.2	76.7	33.7	20.3 (36.6)	7.9	22.5	48.8	12.6	15.3 (39.6)	3.5	13.8	35.3
	Cases	94.3	39.6 (86.2)	18.5	42.0	82.1	40.2	20.0 (33.6)	8.7	22.3	48.4	13.4	16.8 (42.3)	4.7	14.6	39.4
5‐year lag	Controls	93.4	31.6 (59.6)	15.2	35.9	69.1	32.6	18.8 (34.1)	7.5	21.3	43.9	11.8	14.3 (38.0)	3.4	12.0	33.2
	Cases	92.0	35.6 (81.5)	15.9	37.0	75.1	38.2	18.5 (30.9)	8.2	21.2	45.0	11.9	16.4 (42.1)	4.5	13.4	37.3
10‐year lag	Controls	90.5	27.0 (53.0)	12.3	30.3	59.9	30.1	16.7 (30.9)	6.4	19.2	37.5	10.5	12.5 (33.8)	2.9	11.0	28.9
	Cases	88.0	30.7 (73.2)	13.2	30.9	68.8	35.1	16.7 (27.9)	7.1	19.0	39.9	10.1	15.6 (42.0)	3.3	13.5	32.4
1‐ to 4‐year time window	Controls	66.6	4.0 (7.3)	1.9	4.8	9.2	13.5	4.0 (5.6)	2.1	4.5	11.8	5.0	3.3 (5.0)	1.5	3.9	9.7
	Cases	69.9	4.9 (9.1)	2.5	5.1	11.1	18.0	4.0 (6.3)	2.3	4.4	8.8	6.2	3.6 (5.2)	2.0	4.3	8.6
5‐ to 9‐year time window	Controls	73.2	5.6 (10.1)	2.9	6.8	12.8	16.2	5.3 (7.1)	3.0	6.4	15.3	5.9	4.9 (9.3)	1.7	5.8	14.6
	Cases	75.0	6.2 (11.9)	2.9	6.7	14.6	21.0	4.6 (6.8)	2.4	5.6	11.6	6.8	4.5 (7.1)	2.0	4.9	12.1
Time weighted average																
1‐year lag	Controls	94.5	1.5 (2.3)	1.0	1.7	3.1	33.7	0.3 (1.0)	0.1	0.2	0.6	12.6	1.3 (2.5)	0.5	1.5	3.6
	Cases	94.3	1.6 (2.6)	0.9	1.8	3.4	40.2	0.3 (0.9)	0.1	0.2	0.7	13.4	1.4 (2.3)	0.6	1.5	3.9
5‐year lag	Controls	93.4	1.5 (2.4)	0.9	1.7	3.1	32.6	1.5 (2.2)	0.8	1.6	4.0	11.8	1.3 (2.6)	0.5	1.5	3.6
	Cases	92.0	1.6 (2.6)	0.9	1.8	3.4	38.2	1.4 (2.0)	0.7	1.6	4.0	11.9	1.5 (2.4)	0.5	1.5	3.9
10‐year lag	Controls	90.5	1.5 (2.4)	0.9	1.7	3.2	30.1	1.5 (2.1)	0.7	1.6	4.0	10.5	1.3 (2.6)	0.5	1.5	3.6
	Cases	88.0	1.6 (2.8)	0.9	1.8	3.4	35.1	1.4 (1.9)	0.7	1.5	4.0	10.1	1.3 (2.3)	0.5	1.4	3.6
1‐ to 4‐year time window	Controls	66.6	1.5 (2.7)	0.9	1.7	3.6	13.5	1.6 (2.1)	0.9	1.6	4.4	5.0	1.2 (1.7)	0.5	1.5	3.6
	Cases	69.9	1.7 (3.3)	0.9	1.7	3.8	18.0	1.5 (2.6)	0.8	1.6	4.0	6.2	1.4 (2.1)	0.9	1.5	3.7
5‐ to 9‐year time window	Controls	73.2	1.5 (2.7)	0.9	1.7	3.6	16.2	1.6 (2.1)	0.9	1.7	4.4	5.9	1.4 (2.5)	0.5	1.6	3.9
	Cases	75.0	1.7 (3.1)	0.9	1.7	3.7	21.0	1.4 (1.9)	0.8	1.6	4.0	6.8	1.4 (2.2)	0.7	1.5	3.9

*Note*: Electric fields (E) in V/m. Method 1: JEM intensity values applied to all exposed jobs. Method 2: ISCO88 jobs having a prevalence of exposure falling below the median of JEM prevalence values (E fields: 5.1%; H fields: 4.8% for RF and 1.5% for IF‐RF E and H fields) considered nonexposed; JEM intensity values applied to remaining jobs. Method 3: JEM intensity values applied only to jobs where participants reported the use of any RF‐EMF occupational source.

^a^ Percentage of exposed participants based on the total number of participants.

^b^ Arithmetic mean (SD) (exposed participants).

^c^ Percentiles (exposed participants).

**TABLE 2B:** Cumulative and average estimates of exposure based on three methods of attributing RF‐JEM estimates for glioma cases (*n* = 1819) and controls (*n* = 5227).

Exposure period	Participants status	Exposure assessment Method 1					Exposure assessment Method 2					Exposure assessment Method 3				
		%^a^	Mean (SD)^b^	50th^c^	75th^c^	90th^c^	%^a^	Mean (SD)^b^	50th^c^	75th^c^	90th^c^	%^a^	Mean (SD)^b^	50th^c^	75th^c^	90th^c^
Cumulative exposure																
1‐year lag	Controls	94.0	10.2 (33.5)	2.1	5.5	18.0	32.9	5.9 (21.1)	1.1	3.2	10.2	12.3	4.6 (22.1)	0.6	1.8	6.5
	Cases	93.9	13.9 (47.8)	2.1	5.9	21.2	40.0	7.1 (28.1)	1.1	3.4	10.9	13.2	6.0 (26.8)	0.5	2.3	7.6
5‐year lag	Controls	92.8	9.3 (31.2)	1.8	4.9	16.4	31.8	5.5 (19.7)	1.0	3.1	10.0	11.6	4.4 (21.2)	0.5	1.8	5.7
	Cases	91.5	12.6 (44.7)	1.9	5.3	20.1	37.9	6.7 (27.1)	1.0	3.1	10.3	11.8	5.7 (24.8)	0.5	2.0	7.4
10‐year lag	Controls	89.8	8.1 (28.0)	1.5	4.2	14.5	29.8	5.0 (17.7)	0.8	2.8	8.8	10.4	3.9 (20.1)	0.5	1.6	5.1
	Cases	87.3	11.0 (39.9)	1.5	4.5	17.0	34.9	6.1 (25.1)	0.9	2.9	9.6	10.0	5.2 (22.4)	0.3	1.8	6.6
1‐ to 4‐year time window	Controls	65.8	1.1 (3.5)	0.2	0.5	2.0	12.5	1.1 (3.1)	0.2	0.7	1.9	4.9	0.9 (3.0)	0.2	0.5	1.5
	Cases	68.9	1.5 (5.0)	0.2	0.6	2.4	17.2	1.3 (3.6)	0.2	0.8	2.0	6.2	1.6 (4.8)	0.2	0.5	2.8
5‐ to 9‐year time window	Controls	72.1	1.5 (5.1)	0.3	0.7	2.6	15.3	1.4 (4.1)	0.3	0.9	2.4	5.8	1.3 (4.5)	0.3	0.6	1.9
	Cases	74.0	2.1 (6.9)	0.3	0.8	2.8	20.0	1.5 (4.7)	0.3	0.9	2.0	6.8	1.6 (5.6)	0.2	0.6	2.0
Time weighted average																
1‐year lag	Controls	94.0	0.4 (1.2)	0.1	0.2	0.8	32.9	0.4 (1.1)	0.1	0.2	0.8	12.3	0.4 (1.3)	0.1	0.2	0.7
	Cases	93.9	0.5 (1.6)	0.1	0.3	0.8	40.0	0.5 (1.2)	0.1	0.3	0.8	13.2	0.5 (1.7)	0.1	0.2	1.0
5‐year lag	Controls	92.8	0.4 (1.2)	0.1	0.2	0.8	31.8	0.4 (1.1)	0.1	0.3	0.8	11.6	0.4 (1.3)	0.1	0.2	0.7
	Cases	91.5	0.6 (1.6)	0.1	0.2	0.9	37.9	0.5 (1.3)	0.1	0.3	0.8	11.8	0.5 (1.8)	0.1	0.2	0.9
10‐year lag	Controls	89.8	0.4 (1.3)	0.1	0.2	0.8	29.8	0.4 (1.2)	0.1	0.3	0.8	10.4	0.4 (1.4)	0.1	0.1	0.2
	Cases	87.3	0.6 (1.7)	0.1	0.2	0.8	34.9	0.5 (1.3)	0.1	0.2	0.8	10.0	0.5 (1.8)	0.1	0.1	0.8
1‐ to 4‐year time window	Controls	65.8	0.4 (1.3)	0.1	0.2	0.7	12.5	0.4 (1.1)	0.1	0.3	0.8	4.9	0.3 (1.0)	0.1	0.2	0.7
	Cases	68.9	0.6 (1.8)	0.1	0.2	0.8	17.2	0.5 (1.4)	0.1	0.3	0.8	6.2	0.6 (1.8)	0.1	0.2	1.2
5‐ to 9‐year time window	Controls	72.1	0.4 (1.4)	0.1	0.2	0.7	15.3	0.4 (1.1)	0.1	0.3	0.8	5.8	0.4 (1.3)	0.1	0.2	0.7
	Cases	74.0	0.6 (1.9)	0.1	0.2	0.8	20.0	0.5 (1.3)	0.1	0.2	0.7	6.8	0.5 (1.4)	0.1	0.2	0.8

*Note*: Magnetic fields (H) in V/m. Method 1: JEM intensity values applied to all exposed jobs. Method 2: ISCO88 jobs having a prevalence of exposure falling below the median of JEM prevalence values (E fields: 5.1%; H fields: 4.8% for RF and 1.5% for IF‐RF E and H fields) considered nonexposed; JEM intensity values applied to remaining jobs. Method 3: JEM intensity values applied only to jobs where participants reported the use of any RF‐EMF occupational source.

^a^ Percentage of exposed participants based on the total number of participants;

^b^ Arithmetic mean (SD) (exposed participants);

^c^ Percentiles (exposed participants).

The exposed jobs most frequently reported by study subjects included *Secretaries* (ISCO 4115), *Shop salespersons and demonstrators* (ISCO 5220), and *Other office clerks* (ISCO 4190) which made up 5.2%, 4.9%, and 3.6% of total captured occupations, respectively. However, only ~1% of participants reported using any RF‐EMF source in these occupations.

While the majority of participants were considered “ever exposed” applying a 1‐, 5‐, or 10‐year lag preceding the diagnosis/reference date in Method 1, there were fewer exposed participants when excluding jobs having a low probability of exposure (Method 2), and an even lower proportion of exposed participants when considering participant‐reported data on use of any IF‐RF/RF‐EMF occupational source in their jobs (Method 3). Only a small percentage of participants reported using a source among the most prevalent occupations (*see above*). The proportion of exposed participants was also generally lower in the 1‐ to 4‐ and 5‐ to 9‐year exposure time windows before diagnosis/reference date compared to the 1‐, 5‐, and 10‐year lag exposures.

For E fields, glioma cases tended to demonstrate somewhat higher mean cumulative exposure levels than controls for most lags and time windows and exposure assessment methods. Findings were similar for H fields. For meningioma, cases also tended to display higher mean cumulative exposure levels than controls across type of field, lag, and time window. Strong positive correlations (Spearman coefficients ranging from 0.84 to 0.98) between E and H field exposures were observed for each lag and specific time window of exposure.

**FIGURE 1:**
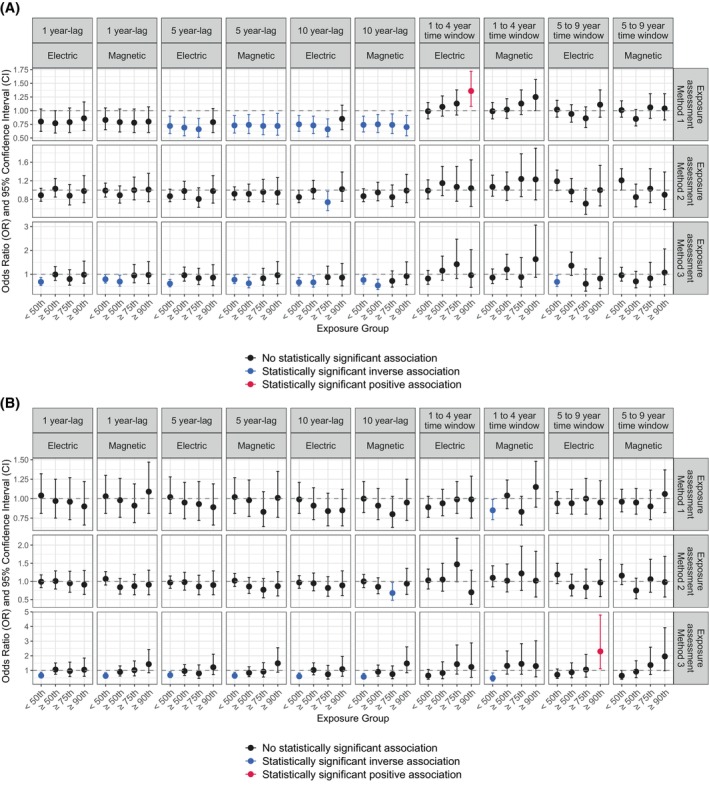
(A) Associations between categories of cumulative exposure to radiofrequency electromagnetic fields (RF‐EMF) based on the 50th, 75th, and 90th percentiles and the risk of gliomas per exposure lag and time window of exposure for Electric (E) and Magnetic (H) fields (Reference group not shown). (B) Associations between categories of cumulative exposure to RF‐EMF based on the 50th, 75th, and 90th percentiles and the risk of meningiomas per exposure lag and time window of exposure for Electric (E) and Magnetic (H) fields (reference group not shown).

Figure 1A,B provides results of the stratified conditional logistic regression analysis of the association between categories of cumulative exposure to IF‐RF/RF‐EMF for E and H fields and the risk of glioma and meningioma, respectively. Additional figures for TWA analyses and detailed results are shown in Figure S1a,b, Tables S2a, S2b, S3a, and S3b, respectively for E and H fields for glioma and meningioma. Results of analysis for TWA exposures to IF‐RF/RF‐EMF are presented in Tables S4a and S4b for glioma and Tables S5a and S5b for meningioma.

Results for the main categorical analysis of glioma using cumulative estimates of exposure indicated mostly no associations for either E or H fields with 1‐, 5‐, or 10‐year lags according to the different exposure assessment approaches. However, some significantly decreased ORs were observed; particularly for E‐fields with exposure assessment Method 1, irrespective of lag time. The only departures from the general pattern of no or inverse associations were found for the 1‐ to 4‐ and 5‐ to 9‐year exposure windows, primarily in the highest exposure groups and for E fields, for both cumulative and average analyses in exposure assessment Methods 1 and 2. For instance, in Method 1, positive associations with gliomas were observed in the highest exposure group of the 1‐ to 4‐year window for both cumulative (OR 1.36; 95% CI 1.08, 1.72) and average analyses (OR 1.27; 95% CI 1.01, 1.59). A positive association was noted in Method 2 for the lowest exposure group with E fields in the 5‐ to 9‐year window (OR 1.22; 95% CI 1.02, 1.47).

Analysis of meningioma generally showed most ORs being close to or <1.0 with no clear trend apparent. Positive ORs were found in the 5‐ to 9‐year window for the highest exposure group with E fields (OR 2.30; 95% CI 1.11, 4.78) and in the 1‐ to 4‐year window for the third exposure group with H fields (OR 2.24; 95% CI 1.21, 4.15), both in Method 3. These results were also based on small numbers of exposed participants, resulting in wide CIs. There was no evidence for a significant trend with all p values for trend being ≥0.05.

Findings from sensitivity analysis for both types of brain tumors did not yield any notable differences from the main findings. There were no clear associations observed for glioma in the analysis which focused on participants who worked the full 4 years in the 1‐ to 4‐year exposure time window. Findings when using the category of <50th percentile of cumulative exposure as the reference group are presented in Tables S6a, S6b, S7a, and S7b.

## 4 DISCUSSION

Associations between occupational exposure to IF‐RF/RF‐EMF and the two most frequent types of brain tumors in adults, glioma and meningioma, were examined applying the INTEROCC RF‐JEM in the INTEROCC study population. Findings using either cumulative or TWA estimates of exposure showed largely null or inverse findings for various lags, specific time windows of exposure, and methods of linking the JEM for both E and H fields. However, some statistically significant positive associations were observed including in the highest exposure categories for glioma for cumulative and TWA exposure in the 1‐ to 4‐year time window for electric fields (E) in the first JEM application method, as well as for meningioma for cumulative exposure in the 5‐ to 9‐year time window for electric fields (E) in the third JEM application method. Some inverse associations were found in the 1‐, 5‐, and 10‐year lag exposures for glioma among Methods 1 and 3 for E and H fields.

The present findings using the RF‐JEM are comparable to a previous analysis of INTEROCC study data with exposure ascertainment based on a SEM‐based approach.^21^ The previous SEM‐based method assigned exposure status and level based on the respondent's reported use of specific occupational RF‐EMF sources. This approach utilized detailed algorithms, taking into account the mean exposure levels of each reported source from the SEM, along with factors including distance to the source, automation, and other modifiers specific to the occupational section. Furthermore, the algorithm considered the frequency of working with or near the source (in hours per day/week) and the duration of exposure (in years), leading to ~10% of participants being classified as exposed to either E‐ or H‐fields. Similarly, in our JEM‐based approach that also takes into account this same participant‐reported source data regarding use of any occupational RF‐EMF source (Method 3), we consistently found no clear associations. Several factors can explain this finding. First, even though Method 3 and the SEM analysis used different exposure estimates, they are strongly correlated. This is because the JEM estimates combine all RF‐EMF source estimates from the SEM for a given job code, and are attributed to nearly the same participant group who reported using a RF‐EMF source. Despite this apparent redundancy, Method 3 was applied in the context of the unique source‐based information contained in the INTEROCC study to assess the combination of job‐ and source‐based information and to compare results with previous SEM analyses. Second, self‐reported information on historical lifelong exposure sources may be affected by information bias, and the extent of misreporting may differ depending on the severity of the disease. In these circumstances, exposure‐disease associations may be under‐estimated, or inflated.^40^ Since both methods rely on participant‐reported data, they are likely to be affected by similar reporting errors. However, it should be noted that the questionnaires in the INTEROCC study were designed to maximize the validity of responses. These questionnaires began with general screening questions about occupational sectors and progressively narrowed down to more detailed queries about specific tasks. This approach attempted to more accurately identify the RF‐EMF sources used at work, rather than directly asking participants for source‐related information. The potential for recall bias, however, makes it difficult to interpret these findings with a high degree of confidence. And finally, there may not be an underlying association between occupational RF exposure and glioma or meningioma risk. Further, reduced ORs, which were also found and discussed in previous INTERPHONE/INTEROCC studies, could be due to participation and selection biases.^41^ Still, the current interpretation of these results is somehow limited by the demographic composition of the INTEROCC study, which underrepresents participants over 60 years of age. This age group is typically at a higher risk for brain tumors, potentially obscuring any potential associations, and impeding the study of effects of long‐latencies. While the JEM and SEM methods may appear redundant, a JEM approach without using self‐reported source data (Methods 1 and 2) is necessary for application in other epidemiologic studies where source‐based information is not typically available.

Method 1 (linking RF intensity estimates to all exposed jobs) and the more specific Method 2 (excluding jobs with a low prevalence of exposure) of linking the JEM to INTEROCC participants occupational history did not rely directly on individualized source‐based information in the attribution of the JEM estimates. The JEM construction method relied on, for each job title reported by one of the nearly 10,000 case and control participants from the INTEROCC study,^26^ attributing the mean SEM exposure values and probability of exposure among all subjects in that job. The SEM was based on a comprehensive collection of measurement data from the literature. While carrying inherent limitations typical of JEMs and a certain degree of uncertainty in the estimates attributed to each occupation,^42^ the data underlying the RF‐JEM is also subject to the potential recall errors mentioned above. There is also some degree of nondifferential misclassification from using the group‐based exposure assignment approach, which will typically not bias the ORs, but will reduce precision. This is known as Berkson‐type error, when group average values are used instead of individual values, thereby reducing the statistical power of a study.^43^ In the construction of the RF‐JEM, levels of exposure were attributed to occupations based only on the exposure of participants who reported using a specific RF‐EMF source in the given occupation, resulting in occupations with exposure level information based on small numbers of exposed participants, and with low prevalences of exposure. Findings from Methods 1 and 2 were however generally consistent.

A few positive associations were observed in the highest exposure category within the 1‐ to 4‐year time window for glioma in Method 1, and for meningioma in Method 3 in the 1‐ to 4‐ and 5‐ to 9‐year time window for E and H fields, respectively, although these findings may have been affected by selection bias. The previous SEM‐based analysis,^21^ described how control participation could be related with socioeconomic status and mobile phone usage, both of which might correlate with occupation and exposure to certain RF‐EMF sources. We also cannot exclude that these positive findings may arise from chance, given the multiple comparisons conducted in this study. Considering the expected improvement of the exposure assessment from Method 1 to 3 here, and the lack of replication of findings regarding glioma in Method 3, this may also further support interpretation of this finding as a chance finding.

Findings from Method 1 should also be interpreted in the context of the very low prevalence of exposure overall across all jobs (median prevalence of 5.1% for E fields, 4.8% for H fields) and the important variability of exposure within job‐codes. In this scenario, many nonexposed participants would be attributed exposure levels (i.e., medical doctors, prevalence of exposure ≈0.7%). Method 2 aimed to mitigate this effect by using the median exposure prevalence across all jobs as a method to reduce potential misclassification bias while preserving statistical power. In studies where the prevalence of exposure is low, it is crucial to maximize specificity to minimize the attenuation of effect estimates due to exposure misclassification.^44^

Other limitations of the current RF‐JEM also need to be considered. The exposure estimates used to create the JEM were drawn from the previous OEMD/SEM databases which relied mostly on source‐based measurement information collected from the available literature from 1974 to 2013, with very few data on personal measurements of RF source exposure.^2^ Personal measurements are generally regarded as a more reliable method to assess exposure to RF‐EMF, and source‐based measurements may suffer several limitations.^45^, ^46^ Exposure‐based information in the JEM was derived from a very low number of workers in most occupations, with fewer than five exposed subjects in 70.4% and 73.0% of ISCO88 codes for E and H fields respectively.^26^ Differences between sexes were also observed regarding occupations and the use of RF‐EMF sources in the INTEROCC dataset. For example, the proportion of male workers was greater among industrial and technical occupations. Additionally, among occupations with high RF‐EMF exposure, females workers tended to report a lesser use of specific occupational RF‐EMF sources compared to males (Table S8). Overall, however, findings remained consistent when examining only male participants (results not shown). Finally, findings from our recent paper^47^ showed that occupational exposure to RF‐EMF among measured jobs could be summarized on the scale of seconds of exposure across a work shift instead of hours, which would also question the relevance of using an 8 h TWA—as is done in the JEM—instead of other metrics such as peak exposure or frequency. These current limitations make the interpretation of our results challenging.

Strengths and limitations of using ICNIRP‐based metrics to estimate cumulative exposure to RF‐EMF have been previously discussed.^21^, ^48^, ^49^, ^50^ ICNIRP ratios are employed to evaluate compliance with regulatory standards based on RF‐EMF thermal effects. However, ICNIRP squared ratios may correlate well with SAR levels, which are also proportional to internal electric fields.^26^ Moreover, in work environments featuring a variety of EMF exposure conditions, the cumulative ICNIRP metric tends to overestimate exposure levels because it is calibrated to worst‐case scenarios rather than average exposures. In our own prior study aimed at measuring personal full‐shift worker exposure across a broad range of occupations, we found that the levels of exposure generally encountered were low (>99% of full‐shift 1 s measurements were <1% ICNIRP 1998 occupational standards) and of short duration.^47^ This type of personal measurement data could be used in future research to improve the reliability of the current RF‐JEM, as well as to assess its performance providing greater insight into the interpretation of our findings in this study.

## 5 CONCLUSION

The previous SEM‐based and the current JEM‐based analyses (with three ways of linking the JEM to participant job histories) produced similar results that fail to show clear trends of associations between various RF‐EMF exposure measures and either glioma or meningioma risk. Nevertheless, interpretation of current findings remains challenging. The method of linking JEM estimates to a job only when RF source use was self‐reported led to significantly lower exposure prevalences and similarly did not suggest clear evidence of increased risk from occupational IF‐RF/RF‐EMF exposures. Findings suggest that the RF‐JEM could potentially be used in studies without self‐reported information available on working with or near RF sources.

While we generally found no associations or inverse associations overall, a few positive associations between exposure to IF‐RF/RF in cumulative and TWA exposure analyses were found with glioma in Method 1 in the highest exposure category in the 1‐ to 4‐year time window of exposure, and with meningioma in Method 3 in the 5‐ to 9‐year window prior to diagnosis/reference date. However, these statistically significant results, not consistent across analysis methods, could be due to chance, and will require verification in other independent studies. Integrating additional criteria when using JEM, such as exposure prevalence similar to Method 2 in this paper, when attributing exposure levels could be considered in further work. Future improvements of the RF‐JEM, including by collecting and integrating personally measured exposure data could be considered.

## AUTHOR CONTRIBUTIONS

**Maxime Turuban:** Conceptualization; data curation; formal analysis; methodology; software; visualization; writing—original draft. **Hans Kromhout:** Conceptualization; supervision; writing—original draft. **Javier Vila:** Supervision; writing—original draft. **Miquel Vallbona‐Vistós:** Data curation; formal analysis; methodology; validation; writing—original draft. **Frank De Vocht:** Supervision; writing—original draft. **Isabelle Baldi:** Writing—review and editing; resources. **Lesley Richardson:** Writing‐review and editing; resources. **Geza Benke:** Writing‐review and editing; resources. **Daniel Krewski:** Writing‐review and editing; resources. **Marie‐Elise Parent:** Writing‐review and editing; resources. **Siegal Sadetzki:** Writing‐review and editing; resources. **Brigitte Schlehofer:** Writing‐review and editing; resources. **Joachim Schüz:** Writing‐review and editing; resources. **Jack Siemiatycki:** Writing‐review and editing; resources. **Martie van Tongeren:** Writing‐review and editing; resources. **Alistair Woodward:** Writing‐review and editing; resources. **Elisabeth Cardis:** Funding acquisition; resources. **Michelle C. Turner:** Conceptualization; funding acquisition; methodology; project administration; supervision; writing—original draft.

## FUNDING INFORMATION

Funding for the OccRF Health Study is provided by Agence Nationale de Sécurité Sanitaire de l'Alimentation, de l'Environnement et du Travail (ANSES) no. EST‐2018 RF‐35. MCT is funded by a Ramón y Cajal fellowship (RYC‐2017‐01892) from the Spanish Ministry of Science, Innovation and Universities and cofunded by the European Social Fund. ISGlobal acknowledges support from the grant CEX2018‐000806‐S funded by MCIN/AEI/10.13039/501100011033, and support from the Generalitat de Catalunya through the CERCA Program. FdV is partly funded by NIHR Applied Research Collaboration West (NIHR ARC West) at the University Hospitals Bristol NHS Foundation Trust. The conduct of the INTEROCC study was funded by the National Institutes for Health (grant no. R01CA124759‐01). The work on the French occupational data was in part funded by AFSSET (Convention no. ST‐2005‐004). The INTERPHONE study was supported by funding from the European Fifth Framework Program, “Quality of Life and Management of Living Resources” (contract 100 QLK4‐CT‐1999901563) and the Union for International Cancer Control (UICC). The UICC received funds for this purpose from the Mobile Manufacturers' Forum (MMF), now Mobile and Wireless Forum (MWF), and the GSM Association. Provision of funds to the INTERPHONE study investigators via the UICC was governed by agreements that guaranteed INTERPHONE's complete scientific independence (http://interphone.iarc.fr/interphone_funding.php). In Australia, funding was received from the Australian National Health and Medical Research Council (EME) (Grant 219129) with funds originally derived from mobile phone service license fees; a University of Sydney Medical Foundation Program; the Cancer Council NSW; and the Cancer Council Victoria. In Montreal, Canada, funding was received from the Canadian Institutes of Health Research (project MOP‐42525); the Canada Research Chairs Program; the Guzzo‐CRS Chair in Environment and Cancer; the Fonds de recherche du Québec – Santé; the Cancer Research Society; in Ottawa and Vancouver, Canada, from the Canadian Institutes of Health Research (CIHR), the latter including partial support from the Canadian Wireless Telecommunications Association; the NSERC/SSHRC/McLaughlin Chair in Population Health Risk Assessment at the University of Ottawa. In France, funding was received by Cancer Research Society (Contract N85142) and three network operators (Orange, SFR, Bouygues Telecom). In Germany, funding was received from the German Mobile Phone Research Program (Deutsches Mobilfunkforschungsprogramm) of the German Federal Ministry for the Environment, Nuclear Safety, and Nature Protection; the Ministry for the Environment and Traffic of the state of Baden‐Wurttemberg; the Ministry for the Environment of the State of North Rhine‐Westphalia; the MAIFOR Program (Mainzer Forschungsforderungsprogramm) of the University of Mainz. In New Zealand, funding was provided by the Health Research Council of New Zealand, Hawkes Bay Medical Research Foundation, the Wellington Medical Research Foundation, the Waikato Medical Research Foundation, and the Cancer Society of New Zealand. Additional funding for the UK study was received from the Mobile Telecommunications, Health and Research (MTHR) program, funding from the Health and Safety Executive, the Department of Health, the UK Network Operators (O2, Orange, T‐Mobile, Vodafone, “3”), and the Scottish Executive. All industry funding was governed by contracts guaranteeing the complete scientific independence of the investigators.

## CONFLICT OF INTEREST STATEMENT

Prof. de Vocht started as ICNIRP commissioner in the summer of 2024. He also conducts consulting for EPRI, not related to this article. Other coauthors have no conflicts of interest to declare.

## ETHICS STATEMENT

Ethics committees at IARC and in the participating countries and regions, including the ethics committee of the Municipal Institute for Medical Investigation (IMIM) in Barcelona, Spain, approved the INTERPHONE and INTEROCC protocol used in the present study. Informed consent of participation was obtained from all participants in INTERPHONE‐INTEROCC studies.

## Supporting information


**Data S1:** Supporting Information.

## Data Availability

The data that support the findings of this study are available from the corresponding author upon reasonable request.

## References

[1] IARC . Non‐ionizing radiation, part 2: radiofrequency electromagnetic fields [Internet]. Accessed March 29, 2023. https://publications.iarc.fr/Book-And-Report-Series/Iarc-Monographs-On-The-Identification-Of-Carcinogenic-Hazards-To-Humans/Non-ionizing-Radiation-Part-2-Radiofrequency-Electromagnetic-Fields-2013 PMC4780878 24772662

[2] Vila J , Bowman JD , Richardson L , et al. A source‐based measurement database for occupational exposure assessment of electromagnetic fields in the INTEROCC study: a literature review approach. Ann Occup Hyg. 2016;60(2):184‐204. 26493616 10.1093/annhyg/mev076 PMC4738235

[3] Vila J , Bowman JD , Figuerola J , et al. Development of a source‐exposure matrix for occupational exposure assessment of electromagnetic fields in the INTEROCC study. J Expo Sci Environ Epidemiol. 2017;27(4):398‐408. 27827378 10.1038/jes.2016.60 PMC5573206

[4] International Commission on Non‐Ionizing Radiation Protection (ICNIRP) . Guidelines for limiting exposure to electromagnetic fields (100 kHz to 300 GHz). Health Phys. 2020;118(5):483‐524. 32167495 10.1097/HP.0000000000001210

[5] Karpowicz J , Ramos V , Gryz K , de Miguel‐Bilbao S . The exposimetric study on the radiofrequency exposures caused by the use of medical devices. 2018 EMF‐Med 1st World Conference on Biomedical Applications of Electromagnetic Fields (EMF‐Med) Split. IEEE; 2018:1‐2.

[6] Stam R , Yamaguchi‐Sekino S . Occupational exposure to electromagnetic fields from medical sources. Ind Health. 2018;56(2):96‐105. 29109357 10.2486/indhealth.2017-0112 PMC5889928

[7] Hansson Mild K , Lundström R , Wilén J . Non‐ionizing radiation in Swedish health care‐exposure and safety aspects. Int J Env Res Public Health. 2019;16(7):1186. 30987016 10.3390/ijerph16071186 PMC6479478

[8] Valic B , Kos B , Gajšek P . Radiofrequency exposures of workers on low‐power FM radio transmitters. Ann Work Expo Health. 2017;61(4):457‐467. 28355437 10.1093/annweh/wxx012

[9] Kaijage Z , Kissaka M . Assessment of radio‐frequency radiation exposure levels: a case of selected Mobile Base stations in Dar es Salaam, Tanzania. IEEE IST‐Africa Week Conference (IST‐Africa). Vol 2018. IEEE; 2018:1‐8.

[10] Politański P , Aniołczyk H , Gadzicka E , Bortkiewicz A , Zmyślony M . Electromagnetic fields exposure assessment among workers at broadcast centers in Poland. Med Pr. 2018;69(5):477‐482. 30245519 10.13075/mp.5893.00685

[11] Xu Y , Zhang X , Chen Y , Ren N , Lin W , Zhang Q . Health effects of electromagnetic fields on reproductive‐age female operators of plastic welding Machines in Fuzhou. China J Occup Env Med. 2016;58(2):148‐153. 26849258 10.1097/JOM.0000000000000581

[12] Zubrzak B , Bieńkowski P , Cała P . Mitigation measures of electromagnetic field exposure in the vicinity of high frequency welders. Med Pr. 2017;68(6):693‐703. 28930303 10.13075/mp.5893.00619

[13] Michalowska J , Tofil A , Jozwik J , Pytka J , Budzynski P , Korzeniewska E . Measurement of high‐frequency electromagnetic fields in CNC machine tools area. 2018 IEEE 4th International Symposium on Wireless Systems within the International Conferences on Intelligent Data Acquisition and Advanced Computing Systems (IDAACS‐SWS). IEEE; 2018:162‐165.

[14] Elliott P , Aresu M , Gao H , et al. Use of TETRA personal radios and sickness absence in the airwave health monitoring study of the British police forces. Environ Res. 2019;175:148‐155. 31125718 10.1016/j.envres.2019.05.012

[15] Gao H , Aresu M , Vergnaud AC , et al. Personal radio use and cancer risks among 48,518 British police officers and staff from the airwave health monitoring study. Br J Cancer. 2019;120(3):375‐378. 30585256 10.1038/s41416-018-0365-6 PMC6354010

[16] Koutsi E , Deligiannis S , Sarantopoulos I , Zarbouti D , Athanasiadou G , Tsoulos G . Radiation measurements in office environment with Wi‐Fi, 3G and 4G users. 2019 8th International Conference on Modern Circuits and Systems Technologies (MOCAST). IEEE; 2019:1‐4.

[17] Massardier‐Pilonchery A , Nerrière E , Croidieu S , et al. Assessment of personal occupational exposure to radiofrequency electromagnetic fields in libraries and media libraries, using calibrated on‐body exposimeters. Int J Env Res Public Health. 2019;16(12):e2087. 10.3390/ijerph16122087 PMC6616949 31200442

[18] Stam R . Occupational exposureto radiofrequency electromagnetic fields. Ind Health. 2022;60:201‐205. 34789598 10.2486/indhealth.2021-0129 PMC9171125

[19] Berrington de González A , Masten SA , Bhatti P , et al. Advisory group recommendations on priorities for the *IARC monographs* . Lancet Oncol. 2024;25(5):546‐548. 38621402 10.1016/S1470-2045(24)00208-0 PMC12640648

[20] Safari Variani A , Saboori S , Shahsavari S , Yari S , Zaroushani V . Effect of occupational exposure to radar radiation on cancer risk: a systematic review and meta‐analysis. Asian Pac J Cancer Prev. 2019;20(11):3211‐3219. 31759343 10.31557/APJCP.2019.20.11.3211 PMC7063007

[21] Vila J , Turner MC , Gracia‐Lavedan E , et al. Occupational exposure to high‐frequency electromagnetic fields and brain tumor risk in the INTEROCC study: an individualized assessment approach. Environ Int. 2018;119:353‐365. 29996112 10.1016/j.envint.2018.06.038 PMC8851381

[22] Groves FD . Cancer in Korean war navy technicians: mortality survey after 40 years. Am J Epidemiol. 2002;155(9):810‐818. 11978584 10.1093/aje/155.9.810

[23] Karipidis KK , Benke G , Sim MR , et al. Occupational exposure to ionizing and non‐ionizing radiation and risk of non‐Hodgkin lymphoma. Int Arch Occup Environ Health. 2007;80(8):663‐670. 17334774 10.1007/s00420-007-0177-0

[24] Szmigielski S . Cancer morbidity in subjects occupationally exposed to high frequency (radiofrequency and microwave) electromagnetic radiation. Sci Total Environ. 1996;180(1):9‐17. 8717316 10.1016/0048-9697(95)04915-0

[25] Tynes T , Hannevik M , Andersen A , Vistnes AI , Haldorsen T . Incidence of breast cancer in Norwegian female radio and telegraph operators. Cancer Causes Control. 1996;7(2):197‐204. 8740732 10.1007/BF00051295

[26] Migault L , Bowman JD , Kromhout H , et al. Development of a job‐exposure matrix for assessment of occupational exposure to high‐frequency electromagnetic fields (3 kHz‐300 GHz). Ann Work Expo Health. 2019;63(9):1013‐1028. 31702767 10.1093/annweh/wxz067 PMC6853656

[27] Klauenberg BJ , Miklavcic D . Radio Frequency Radiation Dosimetry and its Relationship to the Biological Effects of Electromagnetic Fields. Springer Science & Business Media; 2012:577.

[28] Siemiatycki J . Job‐exposure matrices. In: Gail MH , Benichou J , eds. Encyclopedia of Epidemiologic Methods. John Wiley & Sons; 2000:1006.

[29] van Tongeren M , Kincl L , Richardson L , et al. Assessing occupational exposure to chemicals in an international epidemiological study of brain tumours. Ann Occup Hyg. 2013;57(5):610‐626. 23467593 10.1093/annhyg/mes100 PMC3888250

[30] Turner MC , Benke G , Bowman JD , et al. Occupational exposure to extremely low‐frequency magnetic fields and brain tumor risks in the INTEROCC study. Cancer Epidemiol Biomarkers Prev. 2014;23(9):1863‐1872. 24935666 10.1158/1055-9965.EPI-14-0102 PMC4154968

[31] Cardis E , Richardson L , Deltour I , et al. The INTERPHONE study: design, epidemiological methods, and description of the study population. Eur J Epidemiol. 2007;22(9):647‐664. 17636416 10.1007/s10654-007-9152-z

[32] Lavoué J , Pintos J , Tongeren MV , et al. Comparison of exposure estimates in the Finnish job‐exposure matrix FINJEM with a JEM derived from expert assessments performed in Montreal. Occup Environ Med. 2012;69(7):465‐471. 22467796 10.1136/oemed-2011-100154

[33] Lacourt A , Cardis E , Pintos J , et al. INTEROCC case–control study: lack of association between glioma tumors and occupational exposure to selected combustion products, dusts and other chemical agents. BMC Public Health. 2013;13(1):340. 23587105 10.1186/1471-2458-13-340 PMC3637633

[34] McLean D , Fleming S , Turner MC , et al. Occupational solvent exposure and risk of meningioma: results from the INTEROCC multicentre case‐control study. Occup Environ Med. 2014;71(4):253‐258. 24474387 10.1136/oemed-2013-101780

[35] Sadetzki S , Chetrit A , Turner MC , et al. Occupational exposure to metals and risk of meningioma: a multinational case‐control study. J Neurooncol. 2016;130(3):505‐515. 27664150 10.1007/s11060-016-2244-4

[36] Benke G , Turner MC , Fleming S , et al. Occupational solvent exposure and risk of glioma in the INTEROCC study. Br J Cancer. 2017;117(8):1246‐1254. 28910824 10.1038/bjc.2017.285 PMC5674105

[37] Roosli M . Epidemiology of Electromagnetic Fields. CRC Press; 2014:372.

[38] Posit Team . RStudio: Integrated Development Environment for R [Internet]. Posit Software, PBC; 2022 http://www.posit.co/

[39] Therneau TM , Lumley T , Elizabeth A , Cynthia C . Survival: survival analysis [Internet]; 2023 Accessed August 28, 2023. https://cran.r-project.org/web/packages/survival/index.html

[40] Pearce N , Checkoway H , Kriebel D . Bias in occupational epidemiology studies. Occup Environ Med. 2007;64(8):562‐568. 17053019 10.1136/oem.2006.026690 PMC2078501

[41] INTERPHONE Study Group . Brain tumour risk in relation to mobile telephone use: results of the INTERPHONE international case‐control study. Int J Epidemiol. 2010;39(3):675‐694. 20483835 10.1093/ije/dyq079

[42] Sorahan T , Swanson J . Does ‘job’ predict exposure to magnetic fields? Occup Environ Med. 2017;74(12):925. 10.1136/oemed-2017-104494 28611192

[43] Armstrong BG . Effect of measurement error on epidemiological studies of environmental and occupational exposures. Occup Environ Med. 1998;55(10):651‐656. 9930084 10.1136/oem.55.10.651 PMC1757516

[44] Teschke K , Olshan AF , Daniels JL , et al. Occupational exposure assessment in case‐control studies: opportunities for improvement. Occup Environ Med. 2002;59(9):575‐593; discussion 594. 12205230 10.1136/oem.59.9.575 PMC1740358

[45] Bolte JFB . Lessons learnt on biases and uncertainties in personal exposure measurement surveys of radiofrequency electromagnetic fields with exposimeters. Environ Int. 2016;94:724‐735. 27356850 10.1016/j.envint.2016.06.023

[46] Dürrenberger G , Fröhlich J , Röösli M , Mattsson MO . EMF monitoring—concepts, activities, gaps and options. Int J Environ Res Public Health. 2014;11(9):9460‐9479. 25216256 10.3390/ijerph110909460 PMC4199029

[47] Turuban M , Kromhout H , Vila J , Vallbona‐Vistós M , Baldi I , Turner MC . Personal exposure to radiofrequency electromagnetic fields in various occupations in Spain and France. Environ Int. 2023;180:108156. 37722304 10.1016/j.envint.2023.108156

[48] Baste V , Moen BE , Oftedal G , Strand LA , Bjorge L , Hansson MK . Pregnancy outcomes after paternal radiofrequency field exposure aboard fast patrol boats. J Occup Environ Med. 2012;54(4):431‐438. 22354128 10.1097/JOM.0b013e3182445003

[49] Heinrich S , Thomas S , Heumann C , von Kries R , Radon K . Association between exposure to radiofrequency electromagnetic fields assessed by dosimetry and acute symptoms in children and adolescents: a population based cross‐sectional study. Environ Health. 2010;25(9):75. 10.1186/1476-069X-9-75 PMC3006375 21108839

[50] Thomas S , Kühnlein A , Heinrich S , et al. Personal exposure to mobile phone frequencies and well‐being in adults: a cross‐sectional study based on dosimetry. Bioelectromagnetics. 2008;29(6):463‐470. 18393264 10.1002/bem.20414

