# Supplementary material for: Occupational exposure to radiofrequency electromagnetic fields and brain tumor risk: Application of the INTEROCC job‐exposure matrix
Source: Int J Cancer. 2024 Sep 20;156(3):538–51. doi: 10.1002/ijc.35182 (PMC11621992; doi:10.1002/ijc.35182)
Supplement: Supplementary file 1 — Data S1: Supporting Information. [file IJC-156-538-s001.pdf]

# Supplementary material

# Occupational exposure to radiofrequency electromagnetic fields and brain tumor risk: application of the INTEROCC job-exposure matrix

**Authors:** Maxime Turuban, Hans Kromhout, Javier Vila, Miquel Vallbona-Vistós, Frank De Vocht, Isabelle Baldi, Lesley Richardson, Geza Benke, Daniel Krewski, Marie-Elise Parent, Siegal Sadetzki, Brigitte Schlehofer, Joachim Schüz, Jack Siemiatycki, Martie van Tongeren, Alistair Woodward, Elisabeth Cardis, Michelle C Turner

**Table of contents**

Figure S1.a. Associations between categories of average exposure to RF-EMF based on the 50th, 75th and 90th percentiles and the risk of gliomas per exposure lag and time-windows of exposure for Electric (E) and Magnetic (H) fields (Reference group not shown). – Page 1

Figure S1.b. Associations between categories of average exposure to RF-EMF based on the 50th, 75th and 90th percentiles and the risk of meningiomas per exposure lag and time-windows of exposure for Electric (E) and Magnetic (H) fields (Reference group not shown). – Page 2

Table S1.a. Cumulative and average estimates of exposure based on three methods of attributing RF-JEM estimates for meningioma cases (n = 1,758) and controls (n = 5,227). Electric fields (E) in V/m. 5,227). Electric fields (E) in V/m. – Page3

Table S1.b. Cumulative and average estimates of exposure based on three methods of attributing RF-JEM estimates for meningioma cases (n = 1,758) and controls (n = 5,227). Magnetic fields (H) in A/m. – Page 4

Table S2.a. Associations between categories of cumulative exposure to RF-EMF based on the 50th, 75th, and 90th percentiles and the risk of glioma per exposure lag and time-window of exposure. Electric fields (E). – Page5

Table S2.b. Associations between categories of cumulative exposure to RF-EMF based on the 50th, 75th, and 90th percentiles and the risk of glioma per exposure lag and time-window of exposure. Magnetic fields (H). – Page 6

Table S3.a. Associations between categories of cumulative exposure to RF-EMF based on the 50th, 75th, and 90th percentiles and the risk of meningioma per exposure lag and time-window of exposure. Electric fields (E). – Page 7

Table S3.b. Associations between categories of cumulative exposure to RF-EMF based on the 50th, 75th, and 90th percentiles and the risk of meningioma per exposure lag and time-window of exposure. Magnetic fields (H). – Page 8

Table S4.a. Associations between categories of average exposure to RF-EMF based on the 50th, 75th, and 90th percentiles and the risk of glioma per exposure lag and time-window of exposure. Electric fields (E). – Page 9

Table S4.b. Associations between categories of average exposure to RF-EMF based on the 50th, 75th, and 90th percentiles and the risk of glioma per exposure lag and time-window of exposure. Magnetic fields (H). – Page 10

Table S5.a. Associations between categories of average exposure to RF-EMF based on the 50th, 75th, and 90th percentiles and the risk of meningioma per exposure lag and time-window of exposure. Electric fields (E). – Page 11

Table S5.b. Associations between categories of average exposure to RF-EMF based on the 50th, 75th, and 90th percentiles and the risk of meningioma per exposure lag and time-window of exposure. Magnetic fields (H). – Page 12

Table S6.a. Associations between categories of cumulative exposure to RF-EMF based on the 50th, 75th, and 90th percentiles and the risk of glioma per exposure lag and time-window of exposure using < 50th percentile of cumulative exposure as the reference group. Electric fields (E). – Page 13

Table S6.b. Associations between categories of cumulative exposure to RF-EMF based on the 50th, 75th, and 90th percentiles and the risk of glioma per exposure lag and time-window of exposure using < 50th percentile of cumulative exposure as the reference group. Magnetic fields (H). – Page 14

Table S7.a. Associations between categories of cumulative exposure to RF-EMF based on the 50th, 75th, and 90th percentiles and the risk of meningioma per exposure lag and time-window of exposure using < 50th percentile of cumulative exposure as the reference group. Electric fields (E). – Page 15

Table S7.b. Associations between categories of cumulative exposure to RF-EMF based on the 50th, 75th, and 90th percentiles and the risk of meningioma per exposure lag and time-window of exposure using < 50th percentile of cumulative exposure as the reference group. Magnetic fields (H). – Page 16

Table S8. Description of exposure per sex and ISCO88 1-digit occupation across the three methods of linking the INTEROCC RF-JEM to the occupational history of INTEROCC participants for E fields. – Page 17

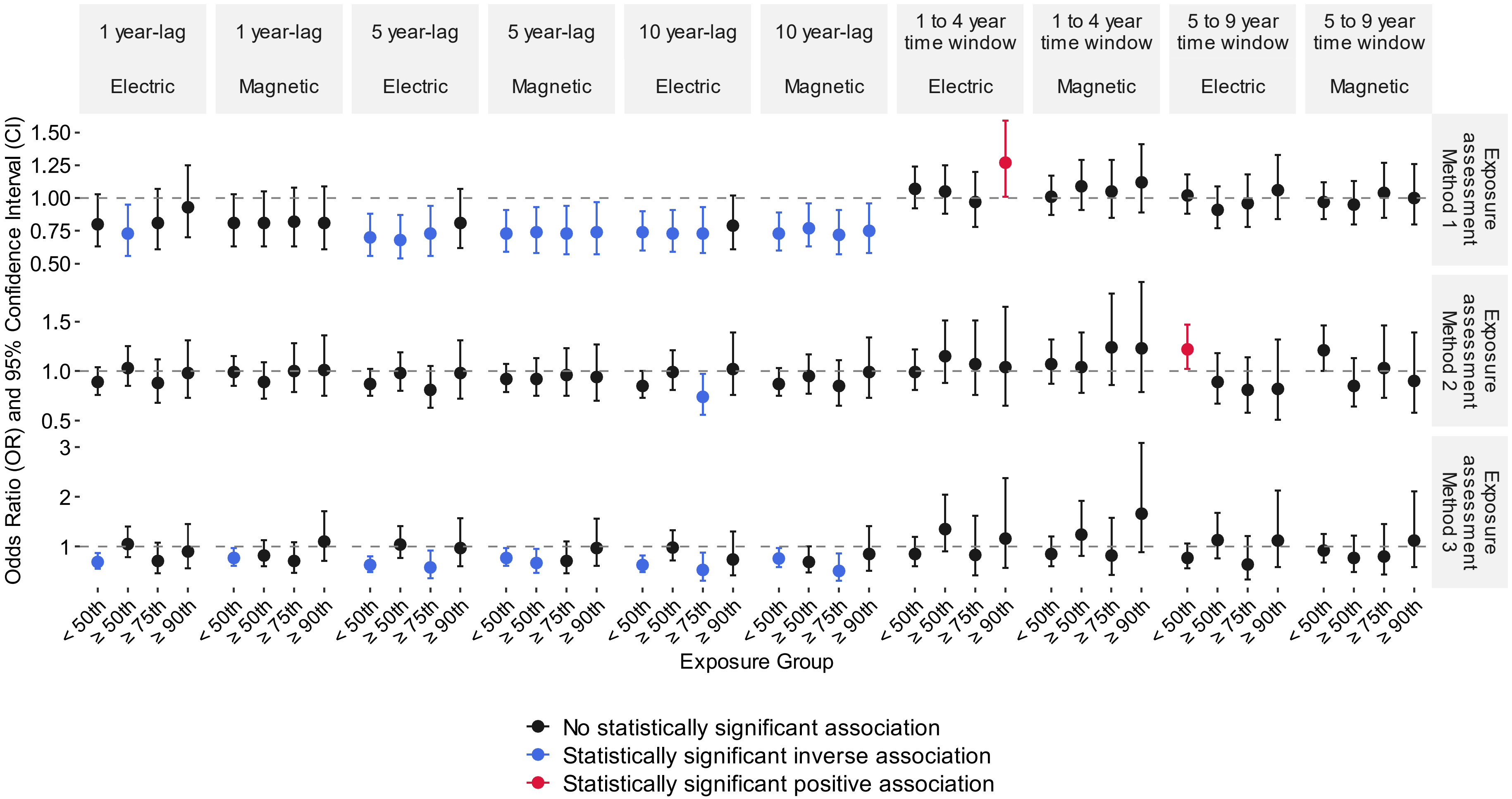


Figure S1.a. Associations between categories of average exposure to RF -EMF based on the 50th, 75th, and 90th percentiles and the risk of gliomas per exposure lag and time-window of exposure for Electric (E) and Magnetic (H) fields (Reference group not shown).

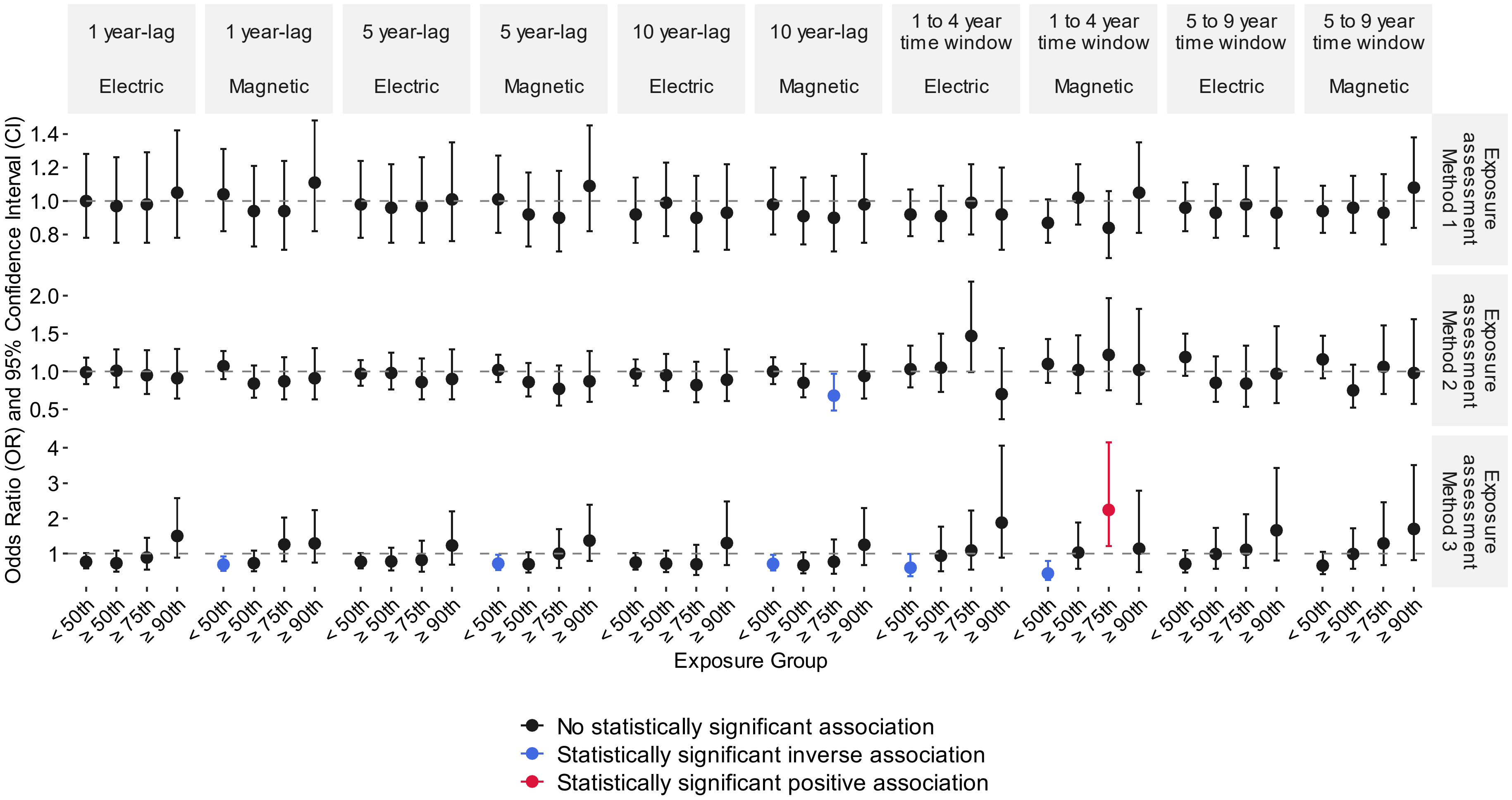


Figure S1.b. Associations between categories of average exposure to RF-EMF based on the 50th, 75th, and 90th percentiles and the risk of meningiomas per exposure lag and time-window of exposure for Electric (E) and Magnetic (H) fields (Reference group not shown).

**Table S1.a** Cumulative and average estimates of exposure based on three methods of attributing RF-JEM estimates for meningioma cases (n = 1,758) and controls (n = 5,227). Electric fields (E) in V/m. 5,227). Electric fields (E) in V/m.

| Exposure period | Participants status | Exposure Assessment Method 1 | | | | | Exposure Assessment Method 2 | | | | | Exposure Assessment Method 3 | | | | |
|---|---|---|---|---|---|---|---|---|---|---|---|---|---|---|---|---|
| | | %[1] | Mean (SD)[2] | 50th[3] | 75th[3] | 90th[3] | %[1] | Mean (SD)[2] | 50th[3] | 75th[3] | 90th[3] | %[1] | Mean (SD)[2] | 50th[3] | 75th[3] | 90th[3] |
| **Cumulative exposure** | | | | | | | | | | | | | | | | |
| 1 year-lag | Controls | 94.5 | 35.1 (64.3) | 17.8 | 40.2 | 76.7 | 33.7 | 20.3 (36.5) | 7.9 | 22.5 | 48.8 | 12.6 | 15.2 (39.5) | 3.6 | 13.5 | 35.3 |
| | Cases | 93.4 | 33.0 (69.4) | 16.6 | 38.6 | 70.8 | 27.2 | 20.2 (65.3) | 7.0 | 21.6 | 47.1 | 8.3 | 18.4 (50.3) | 5.6 | 17.4 | 46.5 |
| 5 year-lag | Controls | 93.3 | 31.6 (59.6) | 15.2 | 35.9 | 68.8 | 32.6 | 18.7 (34.1) | 7.5 | 21.3 | 43.9 | 11.8 | 14.3 (38.0) | 3.4 | 12.0 | 33.3 |
| | Cases | 92.3 | 29.9 (65.4) | 14.2 | 34.1 | 65.7 | 25.7 | 19.0 (60.5) | 6.9 | 20.2 | 43.8 | 7.7 | 17.2 (46.3) | 5.1 | 14.2 | 41.3 |
| 10 year-lag | Controls | 90.4 | 26.9 (53.0) | 12.2 | 30.2 | 59.7 | 30.1 | 16.7 (30.8) | 6.4 | 19.2 | 37.8 | 10.5 | 12.5 (33.9) | 2.9 | 10.6 | 28.0 |
| | Cases | 89.4 | 25.9 (60.6) | 11.7 | 28.7 | 56.1 | 23.8 | 17.2 (54.4) | 5.4 | 18.0 | 37.5 | 6.7 | 14.9 (41.4) | 4.2 | 10.5 | 35.0 |
| 1 to 4 year time window | Controls | 66.8 | 4.0 (7.3) | 1.9 | 4.8 | 9.2 | 13.5 | 4.0 (5.6) | 2.1 | 4.7 | 11.8 | 5.1 | 3.2 (5.0) | 1.4 | 3.8 | 9.4 |
| | Cases | 58.9 | 4.0 (6.6) | 2.6 | 5.0 | 9.2 | 10.2 | 4.4 (8.7) | 2.4 | 4.8 | 11.6 | 3.1 | 4.9 (6.8) | 2.8 | 7.0 | 11.1 |
| 5 to 9 year time window | Controls | 73.2 | 5.6 (10.1) | 2.9 | 6.8 | 12.8 | 16.2 | 5.3 (7.1) | 3.1 | 6.4 | 15.3 | 5.9 | 4.9 (9.3) | 1.8 | 5.8 | 14.6 |
| | Cases | 66.7 | 5.3 (9.3) | 3.3 | 6.8 | 12.3 | 12.1 | 5.3 (11.0) | 2.4 | 6.4 | 15.8 | 3.8 | 7.0 (10.1) | 3.4 | 9.1 | 17.2 |
| **Time weighted average** | | | | | | | | | | | | | | | | |
| 1 year-lag | Controls | 94.5 | 1.5 (2.3) | 1.0 | 1.7 | 3.1 | 33.7 | 0.3 (1.0) | 0.1 | 0.2 | 0.6 | 12.6 | 1.3 (2.5) | 0.5 | 1.5 | 3.6 |
| | Cases | 93.4 | 1.5 (2.1) | 1.0 | 1.7 | 3.0 | 27.2 | 0.4 (1.2) | 0.1 | 0.3 | 0.8 | 8.3 | 1.5 (2.3) | 0.5 | 1.7 | 3.9 |
| 5 year-lag | Controls | 93.3 | 1.5 (2.4) | 0.9 | 1.7 | 3.1 | 32.6 | 1.5 (2.2) | 0.8 | 1.6 | 4.0 | 11.8 | 1.3 (2.6) | 0.5 | 1.5 | 3.6 |
| | Cases | 92.3 | 1.5 (2.2) | 1.0 | 1.7 | 3.1 | 25.7 | 1.6 (2.7) | 0.7 | 1.6 | 4.0 | 7.7 | 1.4 (2.3) | 0.5 | 1.7 | 3.8 |
| 10 year-lag | Controls | 90.4 | 1.5 (2.4) | 0.9 | 1.7 | 3.2 | 30.1 | 1.5 (2.1) | 0.7 | 1.6 | 4.0 | 10.5 | 1.3 (2.6) | 0.5 | 1.5 | 3.6 |
| | Cases | 89.4 | 1.5 (2.2) | 1.0 | 1.7 | 3.1 | 23.8 | 1.5 (2.7) | 0.7 | 1.6 | 3.9 | 6.7 | 1.3 (2.3) | 0.5 | 1.5 | 3.7 |
| 1 to 4 year time window | Controls | 66.8 | 1.5 (2.7) | 0.9 | 1.7 | 3.6 | 13.5 | 1.5 (2.0) | 0.9 | 1.6 | 4.4 | 5.1 | 1.2 (1.7) | 0.5 | 1.5 | 3.7 |
| | Cases | 58.9 | 1.4 (2.3) | 0.9 | 1.7 | 3.2 | 10.2 | 1.7 (3.1) | 0.9 | 1.7 | 4.1 | 3.1 | 2.1 (2.7) | 1.3 | 3.3 | 4.1 |
| 5 to 9 year time window | Controls | 73.2 | 1.5 (2.7) | 0.9 | 1.7 | 3.6 | 16.2 | 1.6 (2.1) | 0.9 | 1.7 | 4.4 | 5.9 | 1.4 (2.5) | 0.5 | 1.6 | 3.9 |
| | Cases | 66.7 | 1.5 (2.5) | 0.9 | 1.7 | 3.2 | 12.1 | 1.5 (2.8) | 0.8 | 1.6 | 3.9 | 3.8 | 1.9 (2.6) | 0.9 | 2.3 | 4.4 |

*[1] Percentage of exposed participants based on the total number of participants; [2] Arithmetic mean (Standard deviation) (exposed participants); [3] Percentiles (exposed participants).*

*Method 1: JEM intensity values applied to all exposed jobs.*

*Method 2: ISCO88 jobs having a prevalence of exposure falling below the median of JEM prevalence values (E fields: 5.1%; H fields: 4.8% for RF and 1.5% for IF-RF E and H fields) considered non-exposed; JEM intensity values applied to remaining jobs.*

*Method 3: JEM intensity values applied only to jobs where participants reported the use of any RF-EMF occupational source.*

**Table S1.b** Cumulative and average estimates of exposure based on three methods of attributing RF-JEM estimates for meningioma cases (n = 1,758) and controls (n = 5,227). Magnetic fields (H) in A/m.

| Exposure period | Participants status | Exposure Assessment Method 1 | | | | | Exposure Assessment Method 2 | | | | | Exposure Assessment Method 3 | | | | |
|---|---|---|---|---|---|---|---|---|---|---|---|---|---|---|---|---|
| | | %[1] | Mean (SD)[2] | 50th[3] | 75th[3] | 90th[3] | %[1] | Mean (SD)[2] | 50th[3] | 75th[3] | 90th[3] | %[1] | Mean (SD)[2] | 50th[3] | 75th[3] | 90th[3] |
| **Cumulative exposure** | | | | | | | | | | | | | | | | |
| 1 year-lag | Controls | 94.0 | 10.2 (33.4) | 2.1 | 5.5 | 17.9 | 32.8 | 5.9 (21.1) | 1.1 | 3.2 | 10.2 | 12.3 | 4.6 (22.0) | 0.6 | 1.8 | 6.5 |
| | Cases | 92.9 | 11.4 (41.8) | 1.9 | 4.6 | 15.7 | 26.7 | 5.8 (26.2) | 0.7 | 2.5 | 9.5 | 8.1 | 8.1 (30.8) | 0.8 | 2.7 | 12.1 |
| 5 year-lag | Controls | 92.8 | 9.3 (31.2) | 1.8 | 4.9 | 16.3 | 31.8 | 5.5 (19.7) | 1.0 | 3.1 | 9.8 | 11.6 | 4.4 (21.2) | 0.5 | 1.8 | 5.7 |
| | Cases | 91.8 | 10.6 (40.0) | 1.6 | 4.1 | 13.6 | 25.3 | 5.6 (24.6) | 0.7 | 2.3 | 8.8 | 7.5 | 8.1 (31.2) | 0.7 | 2.6 | 11.4 |
| 10 year-lag | Controls | 89.7 | 8.1 (28.0) | 1.4 | 4.2 | 14.5 | 29.7 | 5.0 (17.7) | 0.9 | 2.9 | 8.9 | 10.4 | 3.9 (20.0) | 0.5 | 1.6 | 5.1 |
| | Cases | 88.7 | 9.6 (37.7) | 1.3 | 3.3 | 12.2 | 23.4 | 5.2 (22.4) | 0.6 | 1.9 | 8.7 | 6.5 | 7.2 (30.6) | 0.6 | 2.2 | 11.2 |
| 1 to 4 year time window | Controls | 66.0 | 1.1 (3.5) | 0.2 | 0.5 | 2.0 | 12.5 | 1.1 (3.1) | 0.2 | 0.7 | 1.9 | 5.0 | 0.9 (3.0) | 0.2 | 0.5 | 1.4 |
| | Cases | 58.1 | 1.1 (3.5) | 0.2 | 0.5 | 2.1 | 9.6 | 1.1 (3.5) | 0.2 | 0.7 | 1.5 | 3.1 | 0.8 (1.5) | 0.4 | 0.6 | 1.9 |
| 5 to 9 year time window | Controls | 72.2 | 1.5 (5.2) | 0.3 | 0.7 | 2.6 | 15.2 | 1.4 (4.1) | 0.3 | 0.9 | 2.4 | 5.8 | 1.4 (4.7) | 0.3 | 0.6 | 1.9 |
| | Cases | 65.8 | 1.5 (5.3) | 0.3 | 0.7 | 2.6 | 11.3 | 1.4 (4.5) | 0.3 | 0.9 | 1.9 | 3.8 | 3.0 (8.1) | 0.4 | 1.4 | 4.8 |
| **Time weighted average** | | | | | | | | | | | | | | | | |
| 1 year-lag | Controls | 94.0 | 0.4 (1.2) | 0.1 | 0.2 | 0.8 | 32.8 | 0.4 (1.1) | 0.1 | 0.2 | 0.8 | 12.3 | 0.4 (1.3) | 0.1 | 0.2 | 0.7 |
| | Cases | 92.9 | 0.5 (1.4) | 0.1 | 0.2 | 0.7 | 26.7 | 0.4 (1.2) | 0.1 | 0.2 | 0.8 | 8.1 | 0.8 (2.3) | 0.1 | 0.2 | 0.9 |
| 5 year-lag | Controls | 92.8 | 0.4 (1.2) | 0.1 | 0.2 | 0.8 | 31.8 | 0.4 (1.1) | 0.1 | 0.3 | 0.8 | 11.6 | 0.4 (1.3) | 0.1 | 0.2 | 0.7 |
| | Cases | 91.8 | 0.5 (1.4) | 0.1 | 0.2 | 0.8 | 25.3 | 0.4 (1.2) | 0.1 | 0.2 | 0.8 | 7.5 | 0.8 (2.4) | 0.1 | 0.2 | 1.3 |
| 10 year-lag | Controls | 89.7 | 0.4 (1.3) | 0.1 | 0.2 | 0.8 | 29.7 | 0.4 (1.2) | 0.1 | 0.3 | 0.8 | 10.4 | 0.4 (1.4) | 0.1 | 0.2 | 0.7 |
| | Cases | 88.7 | 0.5 (1.4) | 0.1 | 0.2 | 0.7 | 23.4 | 0.5 (1.3) | 0.1 | 0.2 | 0.8 | 6.5 | 0.8 (2.5) | 0.1 | 0.2 | 1.1 |
| 1 to 4 year time window | Controls | 66.0 | 0.4 (1.3) | 0.1 | 0.2 | 0.7 | 12.5 | 0.4 (1.1) | 0.1 | 0.3 | 0.8 | 5.0 | 0.3 (1.0) | 0.1 | 0.2 | 0.7 |
| | Cases | 58.1 | 0.4 (1.2) | 0.1 | 0.2 | 0.7 | 9.6 | 0.4 (1.2) | 0.1 | 0.2 | 0.8 | 3.1 | 0.4 (1.2) | 0.1 | 0.3 | 0.7 |
| 5 to 9 year time window | Controls | 72.2 | 0.4 (1.4) | 0.1 | 0.2 | 0.7 | 15.2 | 0.4 (1.1) | 0.1 | 0.3 | 0.8 | 5.8 | 0.4 (1.3) | 0.1 | 0.2 | 0.7 |
| | Cases | 65.8 | 0.4 (1.3) | 0.1 | 0.2 | 0.7 | 11.3 | 0.4 (1.2) | 0.1 | 0.2 | 0.8 | 3.8 | 0.8 (2.2) | 0.1 | 0.4 | 1.4 |

*[1] Percentage of exposed participants based on the total number of participants; [2] Arithmetic mean (Standard deviation) (exposed participants); [3] Percentiles (exposed participants).*
*Method 1: JEM intensity values applied to all exposed jobs.*
*Method 2: ISCO88 jobs having a prevalence of exposure falling below the median of JEM prevalence values (E fields: 5.1%; H fields: 4.8% for RF and 1.5% for IF-RF E and H fields) considered non-exposed; JEM intensity values applied to remaining jobs.*
*Method 3: JEM intensity values applied only to jobs where participants reported the use of any RF-EMF occupational source.*

**Table S2.a.** Associations between categories of cumulative exposure to RF-EMF based on the 50th, 75th, and 90th percentiles and the risk of glioma per exposure lag and time-window of exposure. Electric fields (E).

| | **Exposure Assessment Method 1** | | | | **Exposure Assessment Method 2** | | | | **Exposure Assessment Method 3** | | |
|---|---|---|---|---|---|---|---|---|---|---|---|
| Exposure categories (ICNIRP squared ratio-years) | Cases (n) | Controls (n) | OR [95% CI] | Exposure categories | Cases (n) | Controls (n) | OR [95% CI] | Exposure categories | Cases (n) | Controls (n) | OR [95% CI] |
| **1 year-lag** | | | | | | | | | | | |
| Non exposed | 104 | 288 | *1.00 (ref.)* | Non exposed | 1088 | 3464 | *1.00 (ref.)* | Non exposed | 1576 | 4571 | *1.00 (ref.)* |
| < 17.9 | 838 | 2470 | 0.79 [0.61; 1.01] | < 7.9 | 339 | 885 | 0.87 [0.75; 1.02] | < 3.5 | 104 | 328 | 0.68 [0.54; 0.87] |
| ≥ 17.9-40.2 | 422 | 1234 | 0.75 [0.58; 0.98] | ≥ 7.9-22.5 | 209 | 437 | 1.02 [0.84; 1.24] | ≥ 3.5-13.8 | 76 | 164 | 1.00 [0.75; 1.33] |
| ≥ 40.2-76.7 | 265 | 741 | 0.79 [0.60; 1.05] | ≥ 22.5-48.8 | 111 | 264 | 0.88 [0.69; 1.13] | ≥ 13.8-35.3 | 34 | 98 | 0.77 [0.51; 1.15] |
| ≥ 76.7 | 188 | 494 | 0.85 [0.63; 1.15] | ≥ 48.8 | 72 | 177 | 0.97 [0.72; 1.30] | ≥ 35.3 | 29 | 66 | 1.00 [0.64; 1.59] |
| **5 year-lag** | | | | | | | | | | | |
| Non exposed | 146 | 346 | *1.00 (ref.)* | Non exposed | 1124 | 3523 | *1.00 (ref.)* | Non exposed | 1603 | 4610 | *1.00 (ref.)* |
| < 15.2 | 821 | 2441 | 0.71 [0.56; 0.88] | < 7.5 | 327 | 852 | 0.86 [0.73; 1.00] | < 3.4 | 90 | 309 | 0.62 [0.48; 0.80] |
| ≥ 15.2-35.9 | 422 | 1220 | 0.68 [0.54; 0.87] | ≥ 7.5-21.3 | 197 | 429 | 0.97 [0.79; 1.18] | ≥ 3.4-12 | 65 | 154 | 0.93 [0.69; 1.27] |
| ≥ 35.9-69.1 | 237 | 732 | 0.65 [0.50; 0.84] | ≥ 21.3-43.9 | 99 | 253 | 0.82 [0.63; 1.06] | ≥ 12-33.2 | 37 | 92 | 0.84 [0.57; 1.25] |
| ≥ 69.1 | 191 | 488 | 0.78 [0.59; 1.03] | ≥ 43.9 | 72 | 170 | 0.95 [0.71; 1.29] | ≥ 33.2 | 24 | 62 | 0.89 [0.54; 1.45] |
| **10 year-lag** | | | | | | | | | | | |
| Non exposed | 219 | 499 | *1.00 (ref.)* | Non exposed | 1181 | 3652 | *1.00 (ref.)* | Non exposed | 1636 | 4676 | *1.00 (ref.)* |
| < 12.3 | 778 | 2364 | 0.73 [0.60; 0.90] | < 6.4 | 299 | 788 | 0.85 [0.72; 1.00] | < 2.9 | 86 | 276 | 0.68 [0.52; 0.88] |
| ≥ 12.3-30.3 | 407 | 1182 | 0.72 [0.57; 0.89] | ≥ 6.4-19.2 | 182 | 394 | 0.97 [0.79; 1.19] | ≥ 2.9-11 | 44 | 137 | 0.66 [0.47; 0.95] |
| ≥ 30.3-59.9 | 223 | 709 | 0.67 [0.52; 0.85] | ≥ 19.2-37.5 | 87 | 235 | 0.76 [0.58; 1.00] | ≥ 11-28.9 | 33 | 83 | 0.87 [0.57; 1.33] |
| ≥ 59.9 | 192 | 473 | 0.83 [0.64; 1.08] | ≥ 37.5 | 70 | 158 | 0.99 [0.73; 1.35] | ≥ 28.9 | 20 | 55 | 0.83 [0.49; 1.42] |
| **1 to 4 year time window** | | | | | | | | | | | |
| Non exposed | 547 | 1744 | *1.00 (ref.)* | Non exposed | 1491 | 4522 | *1.00 (ref.)* | Non exposed | 1706 | 4966 | *1.00 (ref.)* |
| < 1.9 | 580 | 1747 | 0.99 [0.85; 1.15] | < 2.1 | 161 | 355 | 1.02 [0.83; 1.26] | < 1.5 | 46 | 132 | 0.80 [0.56; 1.14] |
| ≥ 1.9-4.8 | 336 | 887 | 1.08 [0.91; 1.29] | ≥ 2.1-4.5 | 90 | 176 | 1.14 [0.87; 1.50] | ≥ 1.5-3.9 | 36 | 64 | 1.17 [0.77; 1.79] |
| ≥ 4.8-9.2 | 201 | 530 | 1.13 [0.92; 1.39] | ≥ 4.5-11.8 | 51 | 108 | 1.11 [0.78; 1.58] | ≥ 3.9-9.7 | 21 | 39 | 1.41 [0.81; 2.45] |
| ≥ 9.2 | 155 | 319 | 1.36 [1.08; 1.72] | ≥ 11.8 | 26 | 66 | 1.00 [0.62; 1.61] | ≥ 9.7 | 10 | 26 | 1.01 [0.47; 2.17] |
| **5 to 9 year time window** | | | | | | | | | | | |
| Non exposed | 454 | 1399 | *1.00 (ref.)* | Non exposed | 1437 | 4378 | *1.00 (ref.)* | Non exposed | 1695 | 4916 | *1.00 (ref.)* |
| < 2.9 | 689 | 1922 | 1.03 [0.88; 1.19] | < 3 | 214 | 425 | 1.20 [0.99; 1.44] | < 1.7 | 50 | 156 | 0.70 [0.50; 0.98] |
| ≥ 2.9-6.8 | 367 | 1053 | 0.95 [0.80; 1.13] | ≥ 3-6.4 | 99 | 221 | 0.97 [0.75; 1.26] | ≥ 1.7-5.8 | 53 | 85 | 1.33 [0.93; 1.90] |
| ≥ 6.8-12.8 | 149 | 471 | 0.85 [0.68; 1.07] | ≥ 6.4-15.3 | 38 | 119 | 0.71 [0.49; 1.04] | ≥ 5.8-14.6 | 13 | 44 | 0.69 [0.36; 1.30] |
| ≥ 12.8 | 160 | 382 | 1.11 [0.88; 1.39] | ≥ 15.3 | 31 | 84 | 0.97 [0.63; 1.49] | ≥ 14.6 | 8 | 26 | 0.70 [0.31; 1.59] |

*Conditional logistic regression models stratified by sex, age (5 year groups), countries and regions and adjusted for education.*
*Method 1: JEM intensity values applied to all exposed jobs.*
*Method 2: ISCO88 jobs having a prevalence of exposure falling below the median of JEM prevalence values (E fields: 5.1%; H fields: 4.8% for RF and 1.5% for IF-RF E and H fields) considered non-exposed; JEM intensity values applied to remaining jobs.*
*Method 3: JEM intensity values applied only to jobs where participants reported the use of any RF-EMF occupational source.*

**Table S2.b.** Associations between categories of cumulative exposure to RF-EMF based on the 50th, 75th, and 90th percentiles and the risk of glioma per exposure lag and time-window of exposure. Magnetic fields (H).

| | **Exposure Assessment Method 1** | | | **Exposure Assessment Method 2** | | | | **Exposure Assessment Method 3** | | | |
|---|---|---|---|---|---|---|---|---|---|---|---|
| Exposure categories (ICNIRP squared ratio-years) | Cases (n) | Controls (n) | OR [95% CI] | Exposure categories | Cases (n) | Controls (n) | OR [95% CI] | Exposure categories | Cases (n) | Controls (n) | OR [95% CI] |
| **1 year-lag** | | | | | | | | | | | |
| Non exposed | 111 | 312 | *1.00 (ref.)* | Non exposed | 1092 | 3509 | *1.00 (ref.)* | Non exposed | 1579 | 4582 | *1.00 (ref.)* |
| < 2.1 | 833 | 2460 | 0.82 [0.64; 1.04] | < 1.1 | 367 | 859 | 0.97 [0.83; 1.13] | < 0.6 | 124 | 323 | 0.81 [0.64; 1.01] |
| ≥ 2.1-5.5 | 425 | 1226 | 0.79 [0.61; 1.02] | ≥ 1.1-3.2 | 167 | 429 | 0.88 [0.72; 1.08] | ≥ 0.6-1.8 | 49 | 161 | 0.67 [0.48; 0.93] |
| ≥ 5.5-18 | 259 | 737 | 0.78 [0.59; 1.02] | ≥ 3.2-10.2 | 118 | 260 | 0.99 [0.78; 1.27] | ≥ 1.8-6.5 | 38 | 96 | 0.96 [0.65; 1.42] |
| ≥ 18 | 191 | 492 | 0.79 [0.59; 1.06] | ≥ 10.2 | 74 | 170 | 1.00 [0.74; 1.34] | ≥ 6.5 | 29 | 65 | 0.98 [0.62; 1.55] |
| **5 year-lag** | | | | | | | | | | | |
| Non exposed | 154 | 376 | *1.00 (ref.)* | Non exposed | 1130 | 3563 | *1.00 (ref.)* | Non exposed | 1605 | 4619 | *1.00 (ref.)* |
| < 1.8 | 807 | 2426 | 0.72 [0.58; 0.90] | < 1 | 339 | 832 | 0.90 [0.77; 1.05] | < 0.5 | 114 | 304 | 0.80 [0.63; 1.01] |
| ≥ 1.8-4.9 | 420 | 1212 | 0.72 [0.57; 0.91] | ≥ 1-3.1 | 170 | 416 | 0.91 [0.74; 1.12] | ≥ 0.5-1.8 | 41 | 152 | 0.57 [0.40; 0.81] |
| ≥ 4.9-16.4 | 250 | 728 | 0.71 [0.55; 0.91] | ≥ 3.1-10 | 110 | 249 | 0.95 [0.74; 1.22] | ≥ 1.8-5.7 | 32 | 91 | 0.84 [0.55; 1.27] |
| ≥ 16.4 | 188 | 485 | 0.71 [0.54; 0.94] | ≥ 10 | 69 | 167 | 0.93 [0.68; 1.26] | ≥ 5.7 | 27 | 61 | 0.97 [0.61; 1.56] |
| **10 year-lag** | | | | | | | | | | | |
| Non exposed | 231 | 534 | *1.00 (ref.)* | Non exposed | 1184 | 3670 | *1.00 (ref.)* | Non exposed | 1637 | 4682 | *1.00 (ref.)* |
| < 1.5 | 764 | 2347 | 0.73 [0.60; 0.89] | < 0.8 | 308 | 779 | 0.86 [0.74; 1.02] | < 0.5 | 99 | 273 | 0.77 [0.60; 0.98] |
| ≥ 1.5-4.2 | 402 | 1173 | 0.73 [0.59; 0.91] | ≥ 0.8-2.8 | 164 | 389 | 0.94 [0.76; 1.16] | ≥ 0.5-1.6 | 33 | 136 | 0.53 [0.35; 0.78] |
| ≥ 4.2-14.5 | 248 | 703 | 0.74 [0.58; 0.94] | ≥ 2.8-8.8 | 93 | 233 | 0.84 [0.64; 1.09] | ≥ 1.6-5.1 | 26 | 82 | 0.74 [0.47; 1.17] |
| ≥ 14.5 | 174 | 470 | 0.69 [0.53; 0.90] | ≥ 8.8 | 69 | 156 | 0.99 [0.73; 1.34] | ≥ 5.1 | 24 | 54 | 0.94 [0.57; 1.55] |
| **1 to 4 year time window** | | | | | | | | | | | |
| Non exposed | 565 | 1788 | *1.00 (ref.)* | Non exposed | 1506 | 4575 | *1.00 (ref.)* | Non exposed | 1707 | 4972 | *1.00 (ref.)* |
| < 0.2 | 581 | 1739 | 0.99 [0.85; 1.15] | < 0.2 | 156 | 327 | 1.08 [0.87; 1.33] | < 0.2 | 47 | 128 | 0.85 [0.60; 1.21] |
| ≥ 0.2-0.5 | 299 | 840 | 1.03 [0.86; 1.23] | ≥ 0.2-0.7 | 75 | 169 | 1.07 [0.80; 1.43] | ≥ 0.2-0.5 | 35 | 64 | 1.21 [0.79; 1.86] |
| ≥ 0.5-2 | 220 | 530 | 1.14 [0.94; 1.40] | ≥ 0.7-1.9 | 50 | 93 | 1.27 [0.88; 1.83] | ≥ 0.5-1.5 | 13 | 37 | 0.89 [0.46; 1.72] |
| ≥ 2 | 154 | 330 | 1.25 [1.00; 1.58] | ≥ 1.9 | 32 | 63 | 1.20 [0.76; 1.87] | ≥ 1.5 | 17 | 26 | 1.61 [0.86; 3.03] |
| **5 to 9 year time window** | | | | | | | | | | | |
| Non exposed | 473 | 1456 | *1.00 (ref.)* | Non exposed | 1455 | 4428 | *1.00 (ref.)* | Non exposed | 1696 | 4922 | *1.00 (ref.)* |
| < 0.3 | 685 | 1896 | 1.02 [0.88; 1.19] | < 0.3 | 208 | 400 | 1.21 [1.00; 1.46] | < 0.3 | 70 | 154 | 0.97 [0.72; 1.31] |
| ≥ 0.3-0.7 | 317 | 1045 | 0.86 [0.72; 1.02] | ≥ 0.3-0.9 | 75 | 204 | 0.86 [0.65; 1.14] | ≥ 0.3-0.6 | 23 | 75 | 0.67 [0.41; 1.08] |
| ≥ 0.7-2.6 | 200 | 473 | 1.08 [0.87; 1.33] | ≥ 0.9-2.4 | 51 | 115 | 1.05 [0.74; 1.49] | ≥ 0.6-1.9 | 16 | 45 | 0.83 [0.46; 1.51] |
| ≥ 2.6 | 144 | 357 | 1.03 [0.82; 1.30] | ≥ 2.4 | 30 | 80 | 0.89 [0.58; 1.39] | ≥ 1.9 | 14 | 31 | 1.07 [0.56; 2.04] |

*Conditional logistic regression models stratified by sex, age (5 year groups), countries and regions and adjusted for education.*
*Method 1: JEM intensity values applied to all exposed jobs.*
*Method 2: ISCO88 jobs having a prevalence of exposure falling below the median of JEM prevalence values (E fields: 5.1%; H fields: 4.8% for RF and 1.5% for IF-RF E and H fields) considered non-exposed; JEM intensity values applied to remaining jobs.*
*Method 3: JEM intensity values applied only to jobs where participants reported the use of any RF-EMF occupational source.*

**Table S3.a.** Associations between categories of cumulative exposure to RF-EMF based on the 50th, 75th, and 90th percentiles and the risk of meningioma per exposure lag and time-window of exposure. Electric fields (E).

| | **Exposure Assessment Method 1** | | | | **Exposure Assessment Method 2** | | | | **Exposure Assessment Method 3** | | |
|---|---|---|---|---|---|---|---|---|---|---|---|
| Exposure categories (ICNIRP squared ratio-years) | Cases (n) | Controls (n) | OR [95% CI] | Exposure categories | Cases (n) | Controls (n) | OR [95% CI] | Exposure categories | Cases (n) | Controls (n) | OR [95% CI] |
| **1 year-lag** | | | | | | | | | | | |
| Non exposed | 116 | 288 | *1.00 (ref.)* | Non exposed | 1280 | 3466 | *1.00 (ref.)* | Non exposed | 1612 | 4571 | *1.00 (ref.)* |
| < 17.8 | 849 | 2470 | 1.04 [0.81; 1.33] | < 7.9 | 252 | 883 | 0.99 [0.83; 1.18] | < 3.6 | 61 | 328 | 0.66 [0.49; 0.89] |
| ≥ 17.8-40.2 | 410 | 1234 | 0.97 [0.75; 1.26] | ≥ 7.9-22.5 | 111 | 438 | 1.02 [0.80; 1.30] | ≥ 3.6-13.5 | 46 | 164 | 1.10 [0.77; 1.56] |
| ≥ 40.2-76.7 | 242 | 741 | 0.97 [0.74; 1.29] | ≥ 22.5-48.8 | 69 | 264 | 0.95 [0.70; 1.29] | ≥ 13.5-35.3 | 21 | 98 | 0.91 [0.55; 1.51] |
| ≥ 76.7 | 141 | 494 | 0.89 [0.66; 1.21] | ≥ 48.8 | 45 | 176 | 0.91 [0.64; 1.31] | ≥ 35.3 | 17 | 66 | 1.07 [0.61; 1.88] |
| **5 year-lag** | | | | | | | | | | | |
| Non exposed | 135 | 348 | *1.00 (ref.)* | Non exposed | 1306 | 3525 | *1.00 (ref.)* | Non exposed | 1622 | 4611 | *1.00 (ref.)* |
| < 15.2 | 833 | 2440 | 1.02 [0.81; 1.28] | < 7.5 | 235 | 851 | 0.96 [0.81; 1.15] | < 3.4 | 61 | 308 | 0.69 [0.51; 0.93] |
| ≥ 15.2-35.9 | 412 | 1219 | 0.95 [0.75; 1.22] | ≥ 7.5-21.3 | 110 | 428 | 0.98 [0.77; 1.26] | ≥ 3.4-12 | 39 | 154 | 0.97 [0.67; 1.42] |
| ≥ 35.9-68.8 | 232 | 732 | 0.93 [0.72; 1.22] | ≥ 21.3-43.9 | 62 | 253 | 0.88 [0.64; 1.20] | ≥ 12-33.3 | 18 | 92 | 0.83 [0.49; 1.43] |
| ≥ 68.8 | 146 | 488 | 0.89 [0.67; 1.19] | ≥ 43.9 | 44 | 170 | 0.90 [0.63; 1.29] | ≥ 33.3 | 18 | 62 | 1.19 [0.68; 2.07] |
| **10 year-lag** | | | | | | | | | | | |
| Non exposed | 187 | 504 | *1.00 (ref.)* | Non exposed | 1339 | 3655 | *1.00 (ref.)* | Non exposed | 1640 | 4678 | *1.00 (ref.)* |
| < 12.2 | 810 | 2362 | 0.99 [0.81; 1.21] | < 6.4 | 218 | 786 | 0.97 [0.81; 1.16] | < 2.9 | 50 | 275 | 0.61 [0.44; 0.85] |
| ≥ 12.2-30.2 | 401 | 1180 | 0.91 [0.73; 1.14] | ≥ 6.4-19.2 | 103 | 394 | 0.97 [0.75; 1.25] | ≥ 2.9-10.6 | 38 | 137 | 1.06 [0.72; 1.56] |
| ≥ 30.2-59.7 | 217 | 708 | 0.84 [0.66; 1.08] | ≥ 19.2-37.8 | 57 | 234 | 0.87 [0.63; 1.20] | ≥ 10.6-28 | 14 | 82 | 0.74 [0.41; 1.35] |
| ≥ 59.7 | 143 | 473 | 0.84 [0.64; 1.11] | ≥ 37.8 | 40 | 158 | 0.85 [0.58; 1.24] | ≥ 28 | 16 | 55 | 1.12 [0.62; 2.03] |
| **1 to 4 year time window** | | | | | | | | | | | |
| Non exposed | 723 | 1733 | *1.00 (ref.)* | Non exposed | 1579 | 4522 | *1.00 (ref.)* | Non exposed | 1704 | 4960 | *1.00 (ref.)* |
| < 1.9 | 478 | 1753 | 0.89 [0.76; 1.03] | < 2.1 | 83 | 355 | 1.02 [0.78; 1.33] | < 1.4 | 22 | 134 | 0.72 [0.44; 1.15] |
| ≥ 1.9-4.8 | 284 | 890 | 0.94 [0.78; 1.12] | ≥ 2.1-4.7 | 43 | 174 | 1.08 [0.75; 1.56] | ≥ 1.4-3.8 | 10 | 66 | 0.71 [0.35; 1.43] |
| ≥ 4.8-9.2 | 179 | 531 | 0.99 [0.81; 1.23] | ≥ 4.7-11.8 | 39 | 109 | 1.44 [0.96; 2.15] | ≥ 3.8-9.4 | 14 | 40 | 1.42 [0.74; 2.73] |
| ≥ 9.2 | 93 | 320 | 0.99 [0.76; 1.30] | ≥ 11.8 | 13 | 67 | 0.70 [0.38; 1.32] | ≥ 9.4 | 8 | 27 | 1.25 [0.54; 2.90] |
| **5 to 9 year time window** | | | | | | | | | | | |
| Non exposed | 585 | 1399 | *1.00 (ref.)* | Non exposed | 1546 | 4380 | *1.00 (ref.)* | Non exposed | 1691 | 4917 | *1.00 (ref.)* |
| < 2.9 | 566 | 1922 | 0.94 [0.81; 1.10] | < 3.1 | 119 | 425 | 1.20 [0.95; 1.52] | < 1.8 | 26 | 155 | 0.71 [0.46; 1.10] |
| ≥ 2.9-6.8 | 353 | 1054 | 0.95 [0.80; 1.13] | ≥ 3.1-6.4 | 46 | 219 | 0.85 [0.60; 1.20] | ≥ 1.8-5.8 | 17 | 85 | 0.88 [0.50; 1.52] |
| ≥ 6.8-12.8 | 148 | 469 | 1.01 [0.81; 1.27] | ≥ 6.4-15.3 | 24 | 120 | 0.84 [0.53; 1.34] | ≥ 5.8-14.6 | 12 | 44 | 1.06 [0.54; 2.09] |
| ≥ 12.8 | 106 | 383 | 0.95 [0.73; 1.22] | ≥ 15.3 | 22 | 83 | 0.97 [0.59; 1.61] | ≥ 14.6 | 12 | 26 | 2.30 [1.11; 4.78] |

*Conditional logistic regression models stratified by sex, age (5 year groups), countries and regions and adjusted for education.*
*Method 1: JEM intensity values applied to all exposed jobs.*
*Method 2: ISCO88 jobs having a prevalence of exposure falling below the median of JEM prevalence values (E fields: 5.1%; H fields: 4.8% for RF and 1.5% for IF-RF E and H fields) considered non-exposed; JEM intensity values applied to remaining jobs.*
*Method 3: JEM intensity values applied only to jobs where participants reported the use of any RF-EMF occupational source.*

**Table S3.b.** Associations between categories of cumulative exposure to RF-EMF based on the 50th, 75th, and 90th percentiles and the risk of meningioma per exposure lag and time-window of exposure. Magnetic fields (H).

| | **Exposure Assessment Method 1** | | | **Exposure Assessment Method 2** | | | | **Exposure Assessment Method 3** | | | |
|---|---|---|---|---|---|---|---|---|---|---|---|
| Exposure categories (ICNIRP squared ratio-years) | Cases (n) | Controls (n) | OR [95% CI] | Exposure categories | Cases (n) | Controls (n) | OR [95% CI] | Exposure categories | Cases (n) | Controls (n) | OR [95% CI] |
| **1 year-lag** | | | | | | | | | | | |
| Non exposed | 124 | 312 | *1.00 (ref.)* | Non exposed | 1289 | 3511 | *1.00 (ref.)* | Non exposed | 1616 | 4582 | *1.00 (ref.)* |
| < 2.1 | 858 | 2458 | 1.03 [0.81; 1.31] | < 1.1 | 270 | 858 | 1.07 [0.90; 1.27] | < 0.6 | 58 | 323 | 0.63 [0.47; 0.86] |
| ≥ 2.1-5.5 | 411 | 1228 | 0.99 [0.77; 1.27] | ≥ 1.1-3.2 | 100 | 429 | 0.85 [0.66; 1.09] | ≥ 0.6-1.8 | 40 | 162 | 0.91 [0.63; 1.32] |
| ≥ 5.5-17.9 | 213 | 737 | 0.91 [0.69; 1.20] | ≥ 3.2-10.2 | 56 | 259 | 0.86 [0.62; 1.18] | ≥ 1.8-6.5 | 23 | 95 | 1.05 [0.64; 1.70] |
| ≥ 17.9 | 152 | 492 | 1.08 [0.80; 1.45] | ≥ 10.2 | 42 | 170 | 0.92 [0.64; 1.32] | ≥ 6.5 | 21 | 65 | 1.46 [0.86; 2.49] |
| **5 year-lag** | | | | | | | | | | | |
| Non exposed | 145 | 377 | *1.00 (ref.)* | Non exposed | 1314 | 3565 | *1.00 (ref.)* | Non exposed | 1626 | 4620 | *1.00 (ref.)* |
| < 1.8 | 854 | 2425 | 1.02 [0.82; 1.27] | < 1 | 252 | 831 | 1.03 [0.86; 1.22] | < 0.5 | 56 | 305 | 0.64 [0.47; 0.87] |
| ≥ 1.8-4.9 | 410 | 1212 | 0.98 [0.77; 1.24] | ≥ 1-3.1 | 100 | 415 | 0.87 [0.67; 1.11] | ≥ 0.5-1.8 | 35 | 150 | 0.87 [0.59; 1.29] |
| ≥ 4.9-16.3 | 202 | 728 | 0.83 [0.64; 1.08] | ≥ 3.1-9.8 | 50 | 249 | 0.76 [0.55; 1.07] | ≥ 1.8-5.7 | 20 | 91 | 0.93 [0.56; 1.55] |
| ≥ 16.3 | 147 | 485 | 1.02 [0.76; 1.35] | ≥ 9.8 | 41 | 167 | 0.90 [0.62; 1.31] | ≥ 5.7 | 21 | 61 | 1.53 [0.89; 2.60] |
| **10 year-lag** | | | | | | | | | | | |
| Non exposed | 198 | 540 | *1.00 (ref.)* | Non exposed | 1346 | 3673 | *1.00 (ref.)* | Non exposed | 1644 | 4684 | *1.00 (ref.)* |
| < 1.4 | 834 | 2344 | 1.01 [0.83; 1.23] | < 0.9 | 234 | 777 | 1.00 [0.83; 1.2] | < 0.5 | 47 | 272 | 0.58 [0.41; 0.81] |
| ≥ 1.4-4.2 | 391 | 1171 | 0.91 [0.73; 1.13] | ≥ 0.9-2.9 | 93 | 388 | 0.85 [0.66; 1.11] | ≥ 0.5-1.6 | 33 | 135 | 0.93 [0.62; 1.41] |
| ≥ 4.2-14.5 | 194 | 703 | 0.80 [0.62; 1.03] | ≥ 2.9-8.9 | 44 | 233 | 0.69 [0.48; 0.98] | ≥ 1.6-5.1 | 16 | 82 | 0.76 [0.43; 1.33] |
| ≥ 14.5 | 141 | 469 | 0.96 [0.73; 1.26] | ≥ 8.9 | 40 | 156 | 0.95 [0.65; 1.38] | ≥ 5.1 | 18 | 54 | 1.52 [0.86; 2.69] |
| **1 to 4 year time window** | | | | | | | | | | | |
| Non exposed | 736 | 1776 | *1.00 (ref.)* | Non exposed | 1589 | 4574 | *1.00 (ref.)* | Non exposed | 1704 | 4966 | *1.00 (ref.)* |
| < 0.2 | 465 | 1742 | 0.85 [0.73; 0.99] | < 0.2 | 87 | 327 | 1.10 [0.85; 1.44] | < 0.2 | 15 | 131 | 0.48 [0.27; 0.83] |
| ≥ 0.2-0.5 | 303 | 846 | 1.04 [0.88; 1.24] | ≥ 0.2-0.7 | 42 | 171 | 1.04 [0.72; 1.50] | ≥ 0.2-0.5 | 18 | 65 | 1.34 [0.76; 2.36] |
| ≥ 0.5-2 | 149 | 533 | 0.83 [0.67; 1.04] | ≥ 0.7-1.9 | 24 | 92 | 1.17 [0.72; 1.91] | ≥ 0.5-1.4 | 13 | 39 | 1.42 [0.73; 2.77] |
| ≥ 2 | 105 | 330 | 1.13 [0.87; 1.47] | ≥ 1.9 | 16 | 63 | 1.04 [0.58; 1.87] | ≥ 1.4 | 8 | 26 | 1.34 [0.57; 3.13] |
| **5 to 9 year time window** | | | | | | | | | | | |
| Non exposed | 601 | 1455 | *1.00 (ref.)* | Non exposed | 1560 | 4430 | *1.00 (ref.)* | Non exposed | 1692 | 4923 | *1.00 (ref.)* |
| < 0.3 | 570 | 1899 | 0.97 [0.83; 1.13] | < 0.3 | 110 | 400 | 1.17 [0.92; 1.48] | < 0.3 | 23 | 152 | 0.62 [0.39; 0.99] |
| ≥ 0.3-0.7 | 349 | 1045 | 0.93 [0.79; 1.10] | ≥ 0.3-0.9 | 38 | 202 | 0.76 [0.52; 1.10] | ≥ 0.3-0.6 | 17 | 78 | 0.99 [0.57; 1.74] |
| ≥ 0.7-2.6 | 130 | 471 | 0.94 [0.74; 1.19] | ≥ 0.9-2.4 | 32 | 115 | 1.05 [0.69; 1.61] | ≥ 0.6-1.9 | 12 | 43 | 1.27 [0.64; 2.50] |
| ≥ 2.6 | 108 | 357 | 1.06 [0.83; 1.37] | ≥ 2.4 | 18 | 80 | 1.00 [0.58; 1.72] | ≥ 1.9 | 14 | 31 | 1.96 [0.98; 3.92] |

*Conditional logistic regression models stratified by sex, age (5 year groups), countries and regions and adjusted for education.*
*Method 1: JEM intensity values applied to all exposed jobs.*
*Method 2: ISCO88 jobs having a prevalence of exposure falling below the median of JEM prevalence values (E fields: 5.1%; H fields: 4.8% for RF and 1.5% for IF-RF E and H fields) considered non-exposed; JEM intensity values applied to remaining jobs.*
*Method 3: JEM intensity values applied only to jobs where participants reported the use of any RF-EMF occupational source.*

**Table S4.a.** Associations between categories of average exposure to RF-EMF based on the 50th, 75th, and 90th percentiles and the risk of glioma per exposure lag and time-window of exposure. Electric fields (E).

| | **Exposure Assessment Method 1** | | | **Exposure Assessment Method 2** | | | | **Exposure Assessment Method 3** | | | |
|---|---|---|---|---|---|---|---|---|---|---|---|
| Exposure categories (ICNIRP squared ratio-years) | Cases (n) | Controls (n) | OR [95% CI] | Exposure categories | Cases (n) | Controls (n) | OR [95% CI] | Exposure categories | Cases (n) | Controls (n) | OR [95% CI] |
| **1 year-lag** | | | | | | | | | | | |
| Non exposed | 104 | 288 | *1.00 (ref.)* | Non exposed | 1088 | 3464 | *1.00 (ref.)* | Non exposed | 1576 | 4571 | *1.00 (ref.)* |
| < 1 | 865 | 2470 | 0.79 [0.62; 1.02] | < 0.1 | 381 | 882 | 0.96 [0.82; 1.12] | < 0.5 | 118 | 350 | 0.71 [0.57; 0.89] |
| ≥ 1-1.7 | 384 | 1234 | 0.72 [0.55; 0.94] | ≥ 0.1-0.2 | 183 | 440 | 0.86 [0.7; 1.05] | ≥ 0.5-1.5 | 70 | 146 | 1.08 [0.80; 1.47] |
| ≥ 1.7-3.1 | 266 | 749 | 0.81 [0.61; 1.07] | ≥ 0.2-0.6 | 91 | 264 | 0.80 [0.62; 1.04] | ≥ 1.5-3.6 | 29 | 95 | 0.69 [0.45; 1.06] |
| ≥ 3.1 | 200 | 486 | 0.92 [0.69; 1.23] | ≥ 0.6 | 76 | 177 | 1.06 [0.79; 1.42] | ≥ 3.6 | 26 | 65 | 0.91 [0.57; 1.47] |
| **5 year-lag** | | | | | | | | | | | |
| Non exposed | 146 | 346 | *1.00 (ref.)* | Non exposed | 1124 | 3523 | *1.00 (ref.)* | Non exposed | 1603 | 4610 | *1.00 (ref.)* |
| < 0.9 | 836 | 2443 | 0.69 [0.55; 0.86] | < 0.8 | 360 | 854 | 0.92 [0.78; 1.07] | < 0.5 | 96 | 311 | 0.64 [0.50; 0.82] |
| ≥ 0.9-1.7 | 390 | 1225 | 0.66 [0.52; 0.84] | ≥ 0.8-1.6 | 176 | 430 | 0.84 [0.68; 1.03] | ≥ 0.5-1.5 | 71 | 152 | 1.04 [0.77; 1.41] |
| ≥ 1.7-3.1 | 256 | 725 | 0.73 [0.56; 0.94] | ≥ 1.6-4 | 91 | 255 | 0.76 [0.59; 0.99] | ≥ 1.5-3.6 | 23 | 92 | 0.56 [0.35; 0.89] |
| ≥ 3.1 | 191 | 488 | 0.80 [0.61; 1.05] | ≥ 4 | 68 | 165 | 1.06 [0.78; 1.43] | ≥ 3.6 | 26 | 62 | 0.98 [0.61; 1.58] |
| **10 year-lag** | | | | | | | | | | | |
| Non exposed | 219 | 499 | *1.00 (ref.)* | Non exposed | 1181 | 3652 | *1.00 (ref.)* | Non exposed | 1636 | 4676 | *1.00 (ref.)* |
| < 0.9 | 804 | 2365 | 0.72 [0.59; 0.89] | < 0.7 | 325 | 788 | 0.90 [0.76; 1.05] | < 0.5 | 89 | 281 | 0.67 [0.52; 0.86] |
| ≥ 0.9-1.7 | 390 | 1182 | 0.72 [0.58; 0.90] | ≥ 0.7-1.6 | 170 | 404 | 0.87 [0.71; 1.07] | ≥ 0.5-1.5 | 59 | 139 | 0.95 [0.69; 1.32] |
| ≥ 1.7-3.2 | 234 | 708 | 0.72 [0.57; 0.92] | ≥ 1.6-4 | 81 | 231 | 0.75 [0.57; 0.99] | ≥ 1.5-3.6 | 18 | 80 | 0.50 [0.30; 0.85] |
| ≥ 3.2 | 172 | 473 | 0.78 [0.61; 1.01] | ≥ 4 | 62 | 152 | 1.02 [0.74; 1.40] | ≥ 3.6 | 17 | 51 | 0.74 [0.42; 1.31] |
| **1 to 4 year time window** | | | | | | | | | | | |
| Non exposed | 547 | 1744 | *1.00 (ref.)* | Non exposed | 1491 | 4522 | *1.00 (ref.)* | Non exposed | 1706 | 4966 | *1.00 (ref.)* |
| < 0.9 | 622 | 1757 | 1.07 [0.92; 1.24] | < 0.9 | 187 | 358 | 1.19 [0.97; 1.45] | < 0.5 | 49 | 134 | 0.84 [0.59; 1.18] |
| ≥ 0.9-1.7 | 326 | 892 | 1.05 [0.88; 1.25] | ≥ 0.9-1.6 | 82 | 182 | 1.00 [0.75; 1.32] | ≥ 0.5-1.5 | 40 | 65 | 1.36 [0.90; 2.05] |
| ≥ 1.7-3.6 | 165 | 486 | 0.97 [0.78; 1.20] | ≥ 1.6-4.4 | 31 | 95 | 0.71 [0.46; 1.08] | ≥ 1.5-3.6 | 12 | 36 | 0.84 [0.43; 1.63] |
| ≥ 3.6 | 159 | 348 | 1.27 [1.01; 1.59] | ≥ 4.4 | 28 | 70 | 1.11 [0.70; 1.77] | ≥ 3.6 | 12 | 26 | 1.16 [0.57; 2.39] |
| **5 to 9 year time window** | | | | | | | | | | | |
| Non exposed | 454 | 1399 | *1.00 (ref.)* | Non exposed | 1437 | 4378 | *1.00 (ref.)* | Non exposed | 1695 | 4916 | *1.00 (ref.)* |
| < 0.9 | 693 | 1924 | 1.03 [0.88; 1.19] | < 0.9 | 231 | 441 | 1.22 [1.02; 1.47] | < 0.5 | 57 | 158 | 0.78 [0.57; 1.08] |
| ≥ 0.9-1.7 | 329 | 987 | 0.93 [0.78; 1.10] | ≥ 0.9-1.7 | 77 | 196 | 0.89 [0.67; 1.18] | ≥ 0.5-1.6 | 39 | 75 | 1.11 [0.74; 1.66] |
| ≥ 1.7-3.6 | 196 | 544 | 0.96 [0.78; 1.18] | ≥ 1.7-4.4 | 50 | 132 | 0.81 [0.58; 1.14] | ≥ 1.6-3.9 | 16 | 53 | 0.67 [0.38; 1.19] |
| ≥ 3.6 | 147 | 373 | 1.06 [0.84; 1.33] | ≥ 4.4 | 24 | 80 | 0.82 [0.51; 1.32] | ≥ 3.9 | 12 | 25 | 1.21 [0.59; 2.50] |

*Conditional logistic regression models stratified by sex, age (5 year groups), countries and regions and adjusted for education.*
*Method 1: JEM intensity values applied to all exposed jobs.*
*Method 2: ISCO88 jobs having a prevalence of exposure falling below the median of JEM prevalence values (E fields: 5.1%; H fields: 4.8% for RF and 1.5% for IF-RF E and H fields) considered non-exposed; JEM intensity values applied to remaining jobs.*
*Method 3: JEM intensity values applied only to jobs where participants reported the use of any RF-EMF occupational source.*

**Table S4.b.** Associations between categories of average exposure to RF-EMF based on the 50th, 75th, and 90th percentiles and the risk of glioma per exposure lag and time-window of exposure. Magnetic fields (H).

| | Exposure Assessment Method 1 | | | Exposure Assessment Method 2 | | | | Exposure Assessment Method 3 | | | |
|---|---|---|---|---|---|---|---|---|---|---|---|
| Exposure categories (ICNIRP squared ratio-years) | Cases (n) | Controls (n) | OR [95% CI] | Exposure categories | Cases (n) | Controls (n) | OR [95% CI] | Exposure categories | Cases (n) | Controls (n) | OR [95% CI] |
| **1 year-lag** | | | | | | | | | | | |
| Non exposed | 111 | 312 | *1.00 (ref.)* | Non exposed | 1092 | 3509 | *1.00 (ref.)* | Non exposed | 1579 | 4582 | *1.00 (ref.)* |
| < 0.1 | 822 | 2458 | 0.80 [0.62; 1.02] | < 0.1 | 373 | 859 | 0.97 [0.83; 1.13] | < 0.1 | 120 | 324 | 0.77 [0.61; 0.97] |
| ≥ 0.1-0.2 | 427 | 1228 | 0.81 [0.62; 1.04] | ≥ 0.1-0.2 | 167 | 429 | 0.88 [0.72; 1.08] | ≥ 0.1-0.2 | 57 | 160 | 0.82 [0.60; 1.13] |
| ≥ 0.2-0.8 | 273 | 737 | 0.81 [0.61; 1.06] | ≥ 0.2-0.8 | 112 | 272 | 0.91 [0.71; 1.17] | ≥ 0.2-0.7 | 31 | 96 | 0.73 [0.48; 1.11] |
| ≥ 0.8 | 186 | 492 | 0.80 [0.60; 1.07] | ≥ 0.8 | 75 | 158 | 1.13 [0.84; 1.52] | ≥ 0.7 | 32 | 65 | 1.12 [0.72; 1.75] |
| **5 year-lag** | | | | | | | | | | | |
| Non exposed | 154 | 376 | *1.00 (ref.)* | Non exposed | 1130 | 3563 | *1.00 (ref.)* | Non exposed | 1605 | 4619 | *1.00 (ref.)* |
| < 0.1 | 806 | 2432 | 0.72 [0.58; 0.89] | < 0.1 | 355 | 832 | 0.93 [0.80; 1.09] | < 0.1 | 114 | 304 | 0.78 [0.62; 0.98] |
| ≥ 0.1-0.2 | 419 | 1206 | 0.73 [0.58; 0.92] | ≥ 0.1-0.3 | 160 | 416 | 0.86 [0.70; 1.06] | ≥ 0.1-0.2 | 45 | 152 | 0.68 [0.48; 0.96] |
| ≥ 0.2-0.8 | 261 | 738 | 0.70 [0.55; 0.90] | ≥ 0.3-0.8 | 104 | 261 | 0.87 [0.68; 1.12] | ≥ 0.2-0.7 | 28 | 91 | 0.69 [0.44; 1.08] |
| ≥ 0.8 | 179 | 475 | 0.73 [0.55; 0.95] | ≥ 0.8 | 70 | 155 | 1.04 [0.76; 1.40] | ≥ 0.7 | 27 | 61 | 1.01 [0.63; 1.61] |
| **10 year-lag** | | | | | | | | | | | |
| Non exposed | 231 | 534 | *1.00 (ref.)* | Non exposed | 1184 | 3670 | *1.00 (ref.)* | Non exposed | 1637 | 4682 | *1.00 (ref.)* |
| < 0.1 | 759 | 2347 | 0.72 [0.59; 0.87] | < 0.1 | 328 | 781 | 0.91 [0.78; 1.07] | < 0.1 | 101 | 273 | 0.77 [0.60; 0.98] |
| ≥ 0.1-0.2 | 413 | 1173 | 0.76 [0.62; 0.94] | ≥ 0.1-0.3 | 149 | 387 | 0.86 [0.69; 1.06] | ≥ 0.1-0.2 | 40 | 136 | 0.68 [0.47; 0.99] |
| ≥ 0.2-0.8 | 242 | 704 | 0.71 [0.56; 0.90] | ≥ 0.3-0.8 | 94 | 239 | 0.83 [0.64; 1.08] | ≥ 0.2-0.7 | 19 | 81 | 0.52 [0.31; 0.87] |
| ≥ 0.8 | 174 | 469 | 0.73 [0.57; 0.94] | ≥ 0.8 | 64 | 150 | 0.96 [0.70; 1.31] | ≥ 0.7 | 22 | 55 | 0.86 [0.52; 1.44] |
| **1 to 4 year time window** | | | | | | | | | | | |
| Non exposed | 565 | 1788 | *1.00 (ref.)* | Non exposed | 1506 | 4575 | *1.00 (ref.)* | Non exposed | 1707 | 4972 | *1.00 (ref.)* |
| < 0.1 | 604 | 1803 | 1.01 [0.87; 1.18] | < 0.1 | 165 | 326 | 1.17 [0.95; 1.44] | < 0.1 | 52 | 130 | 0.90 [0.65; 1.27] |
| ≥ 0.1-0.2 | 309 | 822 | 1.09 [0.91; 1.30] | ≥ 0.1-0.3 | 69 | 167 | 0.97 [0.72; 1.31] | ≥ 0.1-0.2 | 29 | 61 | 1.14 [0.72; 1.80] |
| ≥ 0.2-0.7 | 198 | 470 | 1.06 [0.86; 1.31] | ≥ 0.3-0.8 | 47 | 97 | 1.10 [0.76; 1.58] | ≥ 0.2-0.7 | 13 | 38 | 0.83 [0.43; 1.60] |
| ≥ 0.7 | 143 | 344 | 1.12 [0.89; 1.42] | ≥ 0.8 | 32 | 62 | 1.21 [0.77; 1.89] | ≥ 0.7 | 18 | 26 | 1.64 [0.88; 3.06] |
| **5 to 9 year time window** | | | | | | | | | | | |
| Non exposed | 473 | 1456 | *1.00 (ref.)* | Non exposed | 1455 | 4428 | *1.00 (ref.)* | Non exposed | 1696 | 4922 | *1.00 (ref.)* |
| < 0.1 | 666 | 1946 | 0.98 [0.84; 1.13] | < 0.1 | 211 | 433 | 1.14 [0.95; 1.38] | < 0.1 | 67 | 155 | 0.92 [0.68; 1.24] |
| ≥ 0.1-0.2 | 325 | 946 | 0.96 [0.81; 1.14] | ≥ 0.1-0.3 | 74 | 173 | 0.99 [0.74; 1.32] | ≥ 0.1-0.2 | 25 | 74 | 0.77 [0.48; 1.23] |
| ≥ 0.2-0.7 | 214 | 502 | 1.05 [0.85; 1.29] | ≥ 0.3-0.8 | 46 | 119 | 0.89 [0.62; 1.28] | ≥ 0.2-0.7 | 16 | 45 | 0.81 [0.45; 1.47] |
| ≥ 0.7 | 141 | 377 | 0.99 [0.79; 1.25] | ≥ 0.8 | 33 | 74 | 1.08 [0.70; 1.66] | ≥ 0.7 | 15 | 31 | 1.11 [0.59; 2.10] |

*Conditional logistic regression models stratified by sex, age (5 year groups), countries and regions and adjusted for education.*
*Method 1: JEM intensity values applied to all exposed jobs.*
*Method 2: ISCO88 jobs having a prevalence of exposure falling below the median of JEM prevalence values (E fields: 5.1%; H fields: 4.8% for RF and 1.5% for IF-RF E and H fields) considered non-exposed; JEM intensity values applied to remaining jobs.*
*Method 3: JEM intensity values applied only to jobs where participants reported the use of any RF-EMF occupational source.*

**Table S5.a.** Associations between categories of average exposure to RF-EMF based on the 50th, 75th, and 90th percentiles and the risk of meningioma per exposure lag and time-window of exposure. Electric fields (E).

| | **Exposure Assessment Method 1** | | | **Exposure Assessment Method 2** | | | | **Exposure Assessment Method 3** | | | |
|---|---|---|---|---|---|---|---|---|---|---|---|
| Exposure categories (ICNIRP squared ratio-years) | Cases (n) | Controls (n) | OR [95% CI] | Exposure categories | Cases (n) | Controls (n) | OR [95% CI] | Exposure categories | Cases (n) | Controls (n) | OR [95% CI] |
| **1 year-lag** | | | | | | | | | | | |
| Non exposed | 116 | 288 | *1.00 (ref.)* | Non exposed | 1280 | 3466 | *1.00 (ref.)* | Non exposed | 1612 | 4571 | *1.00 (ref.)* |
| < 1 | 803 | 2470 | 1.01 [0.79; 1.29] | < 0.1 | 244 | 881 | 1.00 [0.84; 1.2] | < 0.5 | 71 | 328 | 0.78 [0.59; 1.04] |
| ≥ 1-1.7 | 442 | 1234 | 0.99 [0.76; 1.27] | ≥ 0.1-0.2 | 112 | 440 | 0.88 [0.69; 1.12] | ≥ 0.5-1.5 | 33 | 168 | 0.73 [0.49; 1.09] |
| ≥ 1.7-3.1 | 242 | 749 | 0.97 [0.73; 1.27] | ≥ 0.2-0.6 | 65 | 264 | 0.93 [0.68; 1.25] | ≥ 1.5-3.6 | 22 | 95 | 0.88 [0.54; 1.45] |
| ≥ 3.1 | 155 | 486 | 1.05 [0.78; 1.42] | ≥ 0.6 | 57 | 176 | 1.24 [0.89; 1.73] | ≥ 3.6 | 20 | 65 | 1.50 [0.88; 2.58] |
| **5 year-lag** | | | | | | | | | | | |
| Non exposed | 135 | 348 | *1.00 (ref.)* | Non exposed | 1306 | 3525 | *1.00 (ref.)* | Non exposed | 1622 | 4611 | *1.00 (ref.)* |
| < 0.9 | 793 | 2442 | 0.99 [0.78; 1.24] | < 0.8 | 238 | 853 | 0.95 [0.79; 1.14] | < 0.5 | 68 | 310 | 0.78 [0.59; 1.04] |
| ≥ 0.9-1.7 | 431 | 1217 | 0.96 [0.75; 1.22] | ≥ 0.8-1.6 | 97 | 423 | 0.91 [0.70; 1.18] | ≥ 0.5-1.5 | 32 | 152 | 0.78 [0.52; 1.18] |
| ≥ 1.7-3.1 | 247 | 732 | 0.97 [0.75; 1.26] | ≥ 1.6-4 | 72 | 261 | 1.04 [0.78; 1.39] | ≥ 1.5-3.6 | 20 | 96 | 0.77 [0.46; 1.28] |
| ≥ 3.1 | 152 | 488 | 1.00 [0.75; 1.33] | ≥ 4 | 45 | 165 | 0.94 [0.66; 1.34] | ≥ 3.6 | 16 | 58 | 1.38 [0.77; 2.49] |
| **10 year-lag** | | | | | | | | | | | |
| Non exposed | 187 | 504 | *1.00 (ref.)* | Non exposed | 1339 | 3655 | *1.00 (ref.)* | Non exposed | 1640 | 4678 | *1.00 (ref.)* |
| < 0.9 | 753 | 2362 | 0.92 [0.75; 1.14] | < 0.7 | 218 | 786 | 0.93 [0.77; 1.12] | < 0.5 | 62 | 280 | 0.77 [0.57; 1.04] |
| ≥ 0.9-1.7 | 447 | 1180 | 0.99 [0.80; 1.24] | ≥ 0.7-1.6 | 97 | 403 | 0.94 [0.73; 1.22] | ≥ 0.5-1.5 | 28 | 138 | 0.73 [0.47; 1.13] |
| ≥ 1.7-3.2 | 224 | 708 | 0.90 [0.70; 1.15] | ≥ 1.6-4 | 64 | 231 | 1.04 [0.77; 1.42] | ≥ 1.5-3.6 | 15 | 80 | 0.70 [0.39; 1.24] |
| ≥ 3.2 | 147 | 473 | 0.93 [0.71; 1.22] | ≥ 4 | 40 | 152 | 0.90 [0.62; 1.31] | ≥ 3.6 | 13 | 51 | 1.31 [0.69; 2.49] |
| **1 to 4 year time window** | | | | | | | | | | | |
| Non exposed | 723 | 1733 | *1.00 (ref.)* | Non exposed | 1579 | 4522 | *1.00 (ref.)* | Non exposed | 1704 | 4960 | *1.00 (ref.)* |
| < 0.9 | 491 | 1760 | 0.92 [0.79; 1.07] | < 0.9 | 92 | 357 | 1.14 [0.88; 1.47] | < 0.5 | 19 | 139 | 0.61 [0.37; 1.00] |
| ≥ 0.9-1.7 | 305 | 900 | 0.93 [0.78; 1.11] | ≥ 0.9-1.6 | 42 | 184 | 0.93 [0.65; 1.34] | ≥ 0.5-1.5 | 13 | 66 | 0.87 [0.47; 1.63] |
| ≥ 1.7-3.6 | 146 | 487 | 0.95 [0.76; 1.19] | ≥ 1.6-4.4 | 31 | 95 | 1.50 [0.96; 2.33] | ≥ 1.5-3.7 | 11 | 35 | 1.23 [0.60; 2.53] |
| ≥ 3.6 | 93 | 347 | 0.93 [0.71; 1.21] | ≥ 4.4 | 14 | 69 | 0.71 [0.38; 1.30] | ≥ 3.7 | 11 | 27 | 1.89 [0.88; 4.06] |
| **5 to 9 year time window** | | | | | | | | | | | |
| Non exposed | 585 | 1399 | *1.00 (ref.)* | Non exposed | 1546 | 4380 | *1.00 (ref.)* | Non exposed | 1691 | 4917 | *1.00 (ref.)* |
| < 0.9 | 563 | 1926 | 0.96 [0.83; 1.12] | < 0.9 | 120 | 440 | 1.16 [0.92; 1.46] | < 0.5 | 25 | 157 | 0.69 [0.44; 1.08] |
| ≥ 0.9-1.7 | 338 | 986 | 0.94 [0.79; 1.11] | ≥ 0.9-1.7 | 46 | 195 | 0.98 [0.69; 1.40] | ≥ 0.5-1.6 | 17 | 75 | 0.99 [0.57; 1.73] |
| ≥ 1.7-3.6 | 170 | 543 | 0.99 [0.80; 1.22] | ≥ 1.7-4.4 | 32 | 132 | 1.08 [0.71; 1.64] | ≥ 1.6-3.9 | 15 | 53 | 1.07 [0.58; 1.97] |
| ≥ 3.6 | 102 | 373 | 0.94 [0.72; 1.21] | ≥ 4.4 | 14 | 80 | 0.59 [0.33; 1.08] | ≥ 3.9 | 10 | 25 | 1.93 [0.88; 4.22] |

*Conditional logistic regression models stratified by sex, age (5 year groups), countries and regions and adjusted for education.*
*Method 1: JEM intensity values applied to all exposed jobs.*
*Method 2: ISCO88 jobs having a prevalence of exposure falling below the median of JEM prevalence values (E fields: 5.1%; H fields: 4.8% for RF and 1.5% for IF-RF E and H fields) considered non-exposed; JEM intensity values applied to remaining jobs.*
*Method 3: JEM intensity values applied only to jobs where participants reported the use of any RF-EMF occupational source.*

**Table S5.b.** Associations between categories of average exposure to RF-EMF based on the 50th, 75th, and 90th percentiles and the risk of meningioma per exposure lag and time-window of exposure. Magnetic fields (H).

| | **Exposure Assessment Method 1** | | | **Exposure Assessment Method 2** | | | | **Exposure Assessment Method 3** | | | |
|---|---|---|---|---|---|---|---|---|---|---|---|
| Exposure categories (ICNIRP squared ratio-years) | Cases (n) | Controls (n) | OR [95% CI] | Exposure categories | Cases (n) | Controls (n) | OR [95% CI] | Exposure categories | Cases (n) | Controls (n) | OR [95% CI] |
| **1 year-lag** | | | | | | | | | | | |
| Non exposed | 124 | 312 | *1.00 (ref.)* | Non exposed | 1289 | 3511 | *1.00 (ref.)* | Non exposed | 1616 | 4582 | *1.00 (ref.)* |
| < 0.1 | 858 | 2458 | 1.04 [0.82; 1.32] | < 0.1 | 263 | 858 | 1.02 [0.85; 1.21] | < 0.1 | 63 | 323 | 0.70 [0.52; 0.94] |
| ≥ 0.1-0.2 | 409 | 1228 | 0.95 [0.74; 1.22] | ≥ 0.1-0.2 | 106 | 429 | 0.88 [0.69; 1.12] | ≥ 0.1-0.2 | 35 | 161 | 0.74 [0.50; 1.09] |
| ≥ 0.2-0.8 | 212 | 737 | 0.94 [0.71; 1.24] | ≥ 0.2-0.8 | 49 | 271 | 0.77 [0.55; 1.08] | ≥ 0.2-0.7 | 25 | 96 | 1.25 [0.78; 2.00] |
| ≥ 0.8 | 155 | 492 | 1.10 [0.82; 1.48] | ≥ 0.8 | 51 | 158 | 1.29 [0.91; 1.82] | ≥ 0.7 | 19 | 65 | 1.33 [0.77; 2.31] |
| **5 year-lag** | | | | | | | | | | | |
| Non exposed | 145 | 377 | *1.00 (ref.)* | Non exposed | 1314 | 3565 | *1.00 (ref.)* | Non exposed | 1626 | 4620 | *1.00 (ref.)* |
| < 0.1 | 851 | 2433 | 1.02 [0.82; 1.27] | < 0.1 | 251 | 831 | 1.00 [0.84; 1.19] | < 0.1 | 62 | 304 | 0.73 [0.54; 0.98] |
| ≥ 0.1-0.2 | 402 | 1204 | 0.92 [0.73; 1.17] | ≥ 0.1-0.3 | 99 | 415 | 0.83 [0.65; 1.07] | ≥ 0.1-0.2 | 34 | 152 | 0.75 [0.50; 1.11] |
| ≥ 0.2-0.8 | 211 | 737 | 0.91 [0.70; 1.18] | ≥ 0.3-0.8 | 48 | 260 | 0.77 [0.55; 1.09] | ≥ 0.2-0.7 | 17 | 90 | 0.90 [0.52; 1.56] |
| ≥ 0.8 | 149 | 476 | 1.08 [0.82; 1.44] | ≥ 0.8 | 46 | 156 | 1.15 [0.80; 1.65] | ≥ 0.7 | 19 | 61 | 1.41 [0.81; 2.45] |
| **10 year-lag** | | | | | | | | | | | |
| Non exposed | 198 | 540 | *1.00 (ref.)* | Non exposed | 1346 | 3673 | *1.00 (ref.)* | Non exposed | 1644 | 4684 | *1.00 (ref.)* |
| < 0.1 | 820 | 2344 | 0.98 [0.80; 1.20] | < 0.1 | 234 | 778 | 0.98 [0.81; 1.17] | < 0.1 | 57 | 280 | 0.71 [0.52; 0.97] |
| ≥ 0.1-0.2 | 396 | 1171 | 0.92 [0.74; 1.14] | ≥ 0.1-0.3 | 94 | 387 | 0.84 [0.65; 1.09] | ≥ 0.1-0.2 | 30 | 128 | 0.77 [0.51; 1.18] |
| ≥ 0.2-0.8 | 205 | 703 | 0.90 [0.70; 1.15] | ≥ 0.3-0.8 | 41 | 239 | 0.71 [0.49; 1.02] | ≥ 0.2-0.7 | 12 | 80 | 0.68 [0.36; 1.28] |
| ≥ 0.8 | 139 | 469 | 0.97 [0.74; 1.27] | ≥ 0.8 | 43 | 150 | 1.10 [0.76; 1.59] | ≥ 0.7 | 15 | 55 | 1.25 [0.68; 2.30] |
| **1 to 4 year time window** | | | | | | | | | | | |
| Non exposed | 736 | 1776 | *1.00 (ref.)* | Non exposed | 1589 | 4574 | *1.00 (ref.)* | Non exposed | 1704 | 4966 | *1.00 (ref.)* |
| < 0.1 | 519 | 1812 | 0.87 [0.75; 1.02] | < 0.1 | 87 | 346 | 1.05 [0.81; 1.37] | < 0.1 | 14 | 132 | 0.44 [0.25; 0.79] |
| ≥ 0.1-0.2 | 282 | 821 | 1.02 [0.85; 1.22] | ≥ 0.1-0.3 | 44 | 149 | 1.24 [0.86; 1.79] | ≥ 0.1-0.2 | 15 | 64 | 1.03 [0.57; 1.88] |
| ≥ 0.2-0.7 | 117 | 473 | 0.83 [0.65; 1.05] | ≥ 0.3-0.8 | 19 | 96 | 0.84 [0.49; 1.42] | ≥ 0.2-0.7 | 18 | 39 | 2.24 [1.21; 4.15] |
| ≥ 0.7 | 104 | 345 | 1.05 [0.81; 1.36] | ≥ 0.8 | 19 | 62 | 1.31 [0.75; 2.27] | ≥ 0.7 | 7 | 26 | 1.14 [0.47; 2.78] |
| **5 to 9 year time window** | | | | | | | | | | | |
| Non exposed | 601 | 1455 | *1.00 (ref.)* | Non exposed | 1560 | 4430 | *1.00 (ref.)* | Non exposed | 1692 | 4923 | *1.00 (ref.)* |
| < 0.1 | 588 | 1951 | 0.95 [0.82; 1.10] | < 0.1 | 119 | 432 | 1.16 [0.92; 1.47] | < 0.1 | 23 | 152 | 0.65 [0.41; 1.03] |
| ≥ 0.1-0.2 | 315 | 944 | 0.97 [0.81; 1.15] | ≥ 0.1-0.3 | 39 | 172 | 0.92 [0.63; 1.34] | ≥ 0.1-0.2 | 18 | 77 | 0.98 [0.57; 1.70] |
| ≥ 0.2-0.7 | 139 | 499 | 0.93 [0.74; 1.17] | ≥ 0.3-0.8 | 19 | 119 | 0.59 [0.35; 0.99] | ≥ 0.2-0.7 | 13 | 44 | 1.29 [0.67; 2.47] |
| ≥ 0.7 | 115 | 378 | 1.08 [0.85; 1.39] | ≥ 0.8 | 21 | 74 | 1.30 [0.77; 2.19] | ≥ 0.7 | 12 | 31 | 1.70 [0.83; 3.51] |

*Conditional logistic regression models stratified by sex, age (5 year groups), countries and regions and adjusted for education.*
*Method 1: JEM intensity values applied to all exposed jobs.*
*Method 2: ISCO88 jobs having a prevalence of exposure falling below the median of JEM prevalence values (E fields: 5.1%; H fields: 4.8% for RF and 1.5% for IF-RF E and H fields) considered non-exposed; JEM intensity values applied to remaining jobs.*
*Method 3: JEM intensity values applied only to jobs where participants reported the use of any RF-EMF occupational source.*

**Table S6.a.** Associations between categories of cumulative exposure to RF-EMF based on the 50th, 75th, and 90th percentiles and the risk of glioma per exposure lag and time-window of exposure using < 50th percentile of cumulative exposure as the reference group. Electric fields (E).

| Exposure categories (ICNIRP squared ratio-years) | Exposure Assessment Method 1 | | | Exposure Assessment Method 2 | | | | Exposure Assessment Method 3 | | | |
|---|---|---|---|---|---|---|---|---|---|---|---|
| | Cases (n) | Controls (n) | OR [95% CI] | Exposure categories | Cases (n) | Controls (n) | OR [95% CI] | Exposure categories | Cases (n) | Controls (n) | OR [95% CI] |
| **1 year-lag** | | | | | | | | | | | |
| < 17.9 | 838 | 2470 | *1.00 (ref.)* | < 7.9 | 339 | 885 | *1.00 (ref.)* | < 3.5 | 104 | 328 | *1.00 (ref.)* |
| ≥ 17.9-40.2 | 422 | 1234 | 0.79 [0.61; 1.01] | ≥ 7.9-22.5 | 209 | 437 | 1.17 [0.94; 1.47] | ≥ 3.5-13.8 | 76 | 164 | 0.68 [0.54; 0.87] |
| ≥ 40.2-76.7 | 265 | 741 | 0.75 [0.58; 0.98] | ≥ 22.5-48.8 | 111 | 264 | 1.03 [0.79; 1.36] | ≥ 13.8-35.3 | 34 | 98 | 1.00 [0.75; 1.33] |
| ≥ 76.7 | 188 | 494 | 0.79 [0.60; 1.05] | ≥ 48.8 | 72 | 177 | 1.10 [0.79; 1.52] | ≥ 35.3 | 29 | 66 | 0.77 [0.51; 1.15] |
| **5 year-lag** | | | | | | | | | | | |
| < 15.2 | 821 | 2441 | *1.00 (ref.)* | < 7.5 | 327 | 852 | *1.00 (ref.)* | < 3.4 | 90 | 309 | *1.00 (ref.)* |
| ≥ 15.2-35.9 | 422 | 1220 | 0.71 [0.56; 0.88] | ≥ 7.5-21.3 | 197 | 429 | 1.15 [0.92; 1.44] | ≥ 3.4-12 | 65 | 154 | 0.62 [0.48; 0.80] |
| ≥ 35.9-69.1 | 237 | 732 | 0.68 [0.54; 0.87] | ≥ 21.3-43.9 | 99 | 253 | 0.99 [0.75; 1.32] | ≥ 12-33.2 | 37 | 92 | 0.93 [0.69; 1.27] |
| ≥ 69.1 | 191 | 488 | 0.65 [0.50; 0.84] | ≥ 43.9 | 72 | 170 | 1.12 [0.80; 1.55] | ≥ 33.2 | 24 | 62 | 0.84 [0.57; 1.25] |
| **10 year-lag** | | | | | | | | | | | |
| < 12.3 | 778 | 2364 | *1.00 (ref.)* | < 6.4 | 299 | 788 | *1.00 (ref.)* | < 2.9 | 86 | 276 | *1.00 (ref.)* |
| ≥ 12.3-30.3 | 407 | 1182 | 0.73 [0.60; 0.90] | ≥ 6.4-19.2 | 182 | 394 | 1.21 [0.95; 1.54] | ≥ 2.9-11 | 44 | 137 | 0.68 [0.52; 0.88] |
| ≥ 30.3-59.9 | 223 | 709 | 0.72 [0.57; 0.89] | ≥ 19.2-37.5 | 87 | 235 | 0.95 [0.70; 1.28] | ≥ 11-28.9 | 33 | 83 | 0.66 [0.47; 0.95] |
| ≥ 59.9 | 192 | 473 | 0.67 [0.52; 0.85] | ≥ 37.5 | 70 | 158 | 1.16 [0.83; 1.64] | ≥ 28.9 | 20 | 55 | 0.87 [0.57; 1.33] |
| **1 to 4 year time window** | | | | | | | | | | | |
| < 1.9 | 580 | 1747 | *1.00 (ref.)* | < 2.1 | 161 | 355 | *1.00 (ref.)* | < 1.5 | 46 | 132 | *1.00 (ref.)* |
| ≥ 1.9-4.8 | 336 | 887 | 0.99 [0.85; 1.15] | ≥ 2.1-4.5 | 90 | 176 | 1.20 [0.84; 1.71] | ≥ 1.5-3.9 | 36 | 64 | 0.80 [0.56; 1.14] |
| ≥ 4.8-9.2 | 201 | 530 | 1.08 [0.91; 1.29] | ≥ 4.5-11.8 | 51 | 108 | 1.01 [0.66; 1.55] | ≥ 3.9-9.7 | 21 | 39 | 1.17 [0.77; 1.79] |
| ≥ 9.2 | 155 | 319 | 1.13 [0.92; 1.39] | ≥ 11.8 | 26 | 66 | 0.88 [0.51; 1.51] | ≥ 9.7 | 10 | 26 | 1.41 [0.81; 2.45] |
| **5 to 9 year time window** | | | | | | | | | | | |
| < 2.9 | 689 | 1922 | *1.00 (ref.)* | < 3 | 214 | 425 | *1.00 (ref.)* | < 1.7 | 50 | 156 | *1.00 (ref.)* |
| ≥ 2.9-6.8 | 367 | 1053 | 1.03 [0.88; 1.19] | ≥ 3-6.4 | 99 | 221 | 0.8 [0.57; 1.11] | ≥ 1.7-5.8 | 53 | 85 | 0.70 [0.50; 0.98] |
| ≥ 6.8-12.8 | 149 | 471 | 0.95 [0.80; 1.13] | ≥ 6.4-15.3 | 38 | 119 | 0.59 [0.38; 0.91] | ≥ 5.8-14.6 | 13 | 44 | 1.33 [0.93; 1.90] |
| ≥ 12.8 | 160 | 382 | 0.85 [0.68; 1.07] | ≥ 15.3 | 31 | 84 | 0.81 [0.50; 1.33] | ≥ 14.6 | 8 | 26 | 0.69 [0.36; 1.30] |

*Conditional logistic regression models stratified by sex, age (5 year groups), countries and regions and adjusted for education.*
*Method 1: JEM intensity values applied to all exposed jobs.*
*Method 2: ISCO88 jobs having a prevalence of exposure falling below the median of JEM prevalence values (E fields: 5.1%; H fields: 4.8% for RF and 1.5% for IF-RF E and H fields) considered non-exposed; JEM intensity values applied to remaining jobs.*
*Method 3: JEM intensity values applied only to jobs where participants reported the use of any RF-EMF occupational source.*

**Table S6.b.** Associations between categories of cumulative exposure to RF-EMF based on the 50th, 75th, and 90th percentiles and the risk of glioma per exposure lag and time-window of exposure using < 50th percentile of cumulative exposure as the reference group. Magnetic fields (H).

| Exposure categories (ICNIRP squared ratio-years) | **Exposure Assessment Method 1** | | | **Exposure Assessment Method 2** | | | | **Exposure Assessment Method 3** | | | |
|---|---|---|---|---|---|---|---|---|---|---|---|
| | Cases (n) | Controls (n) | OR [95% CI] | Exposure categories | Cases (n) | Controls (n) | OR [95% CI] | Exposure categories | Cases (n) | Controls (n) | OR [95% CI] |
| **1 year-lag** | | | | | | | | | | | |
| < 2.1 | 833 | 2460 | *1.00 (ref.)* | < 1.1 | 367 | 859 | *1.00 (ref.)* | < 0.6 | 124 | 323 | *1.00 (ref.)* |
| ≥ 2.1-5.5 | 425 | 1226 | 0.82 [0.64; 1.04] | ≥ 1.1-3.2 | 167 | 429 | 0.91 [0.72; 1.14] | ≥ 0.6-1.8 | 49 | 161 | 0.81 [0.64; 1.01] |
| ≥ 5.5-18 | 259 | 737 | 0.79 [0.61; 1.02] | ≥ 3.2-10.2 | 118 | 260 | 1.03 [0.78; 1.35] | ≥ 1.8-6.5 | 38 | 96 | 0.67 [0.48; 0.93] |
| ≥ 18 | 191 | 492 | 0.78 [0.59; 1.02] | ≥ 10.2 | 74 | 170 | 1.00 [0.72; 1.38] | ≥ 6.5 | 29 | 65 | 0.96 [0.65; 1.42] |
| **5 year-lag** | | | | | | | | | | | |
| < 1.8 | 807 | 2426 | *1.00 (ref.)* | < 1 | 339 | 832 | *1.00 (ref.)* | < 0.5 | 114 | 304 | *1.00 (ref.)* |
| ≥ 1.8-4.9 | 420 | 1212 | 0.72 [0.58; 0.90] | ≥ 1-3.1 | 170 | 416 | 0.99 [0.79; 1.25] | ≥ 0.5-1.8 | 41 | 152 | 0.80 [0.63; 1.01] |
| ≥ 4.9-16.4 | 250 | 728 | 0.72 [0.57; 0.91] | ≥ 3.1-10 | 110 | 249 | 1.04 [0.79; 1.38] | ≥ 1.8-5.7 | 32 | 91 | 0.57 [0.40; 0.81] |
| ≥ 16.4 | 188 | 485 | 0.71 [0.55; 0.91] | ≥ 10 | 69 | 167 | 1.01 [0.72; 1.41] | ≥ 5.7 | 27 | 61 | 0.84 [0.55; 1.27] |
| **10 year-lag** | | | | | | | | | | | |
| < 1.5 | 764 | 2347 | *1.00 (ref.)* | < 0.8 | 308 | 779 | *1.00 (ref.)* | < 0.5 | 99 | 273 | *1.00 (ref.)* |
| ≥ 1.5-4.2 | 402 | 1173 | 0.73 [0.60; 0.89] | ≥ 0.8-2.8 | 164 | 389 | 1.11 [0.87; 1.41] | ≥ 0.5-1.6 | 33 | 136 | 0.77 [0.60; 0.98] |
| ≥ 4.2-14.5 | 248 | 703 | 0.73 [0.59; 0.91] | ≥ 2.8-8.8 | 93 | 233 | 0.97 [0.72; 1.30] | ≥ 1.6-5.1 | 26 | 82 | 0.53 [0.35; 0.78] |
| ≥ 14.5 | 174 | 470 | 0.74 [0.58; 0.94] | ≥ 8.8 | 69 | 156 | 1.11 [0.79; 1.57] | ≥ 5.1 | 24 | 54 | 0.74 [0.47; 1.17] |
| **1 to 4 year time window** | | | | | | | | | | | |
| < 0.2 | 581 | 1739 | *1.00 (ref.)* | < 0.2 | 156 | 327 | *1.00 (ref.)* | < 0.2 | 47 | 128 | *1.00 (ref.)* |
| ≥ 0.2-0.5 | 299 | 840 | 0.99 [0.85; 1.15] | ≥ 0.2-0.7 | 75 | 169 | 0.86 [0.58; 1.26] | ≥ 0.2-0.5 | 35 | 64 | 0.85 [0.60; 1.21] |
| ≥ 0.5-2 | 220 | 530 | 1.03 [0.86; 1.23] | ≥ 0.7-1.9 | 50 | 93 | 1.23 [0.80; 1.89] | ≥ 0.5-1.5 | 13 | 37 | 1.21 [0.79; 1.86] |
| ≥ 2 | 154 | 330 | 1.14 [0.94; 1.40] | ≥ 1.9 | 32 | 63 | 1.10 [0.66; 1.85] | ≥ 1.5 | 17 | 26 | 0.89 [0.46; 1.72] |
| **5 to 9 year time window** | | | | | | | | | | | |
| < 0.3 | 685 | 1896 | *1.00 (ref.)* | < 0.3 | 208 | 400 | *1.00 (ref.)* | < 0.3 | 70 | 154 | *1.00 (ref.)* |
| ≥ 0.3-0.7 | 317 | 1045 | 1.02 [0.88; 1.19] | ≥ 0.3-0.9 | 75 | 204 | 0.73 [0.52; 1.02] | ≥ 0.3-0.6 | 23 | 75 | 0.97 [0.72; 1.31] |
| ≥ 0.7-2.6 | 200 | 473 | 0.86 [0.72; 1.02] | ≥ 0.9-2.4 | 51 | 115 | 0.85 [0.56; 1.28] | ≥ 0.6-1.9 | 16 | 45 | 0.67 [0.41; 1.08] |
| ≥ 2.6 | 144 | 357 | 1.08 [0.87; 1.33] | ≥ 2.4 | 30 | 80 | 0.82 [0.50; 1.34] | ≥ 1.9 | 14 | 31 | 0.83 [0.46; 1.51] |

*Conditional logistic regression models stratified by sex, age (5 year groups), countries and regions and adjusted for education.*
*Method 1: JEM intensity values applied to all exposed jobs.*
*Method 2: ISCO88 jobs having a prevalence of exposure falling below the median of JEM prevalence values (E fields: 5.1%; H fields: 4.8% for RF and 1.5% for IF-RF E and H fields) considered non-exposed; JEM intensity values applied to remaining jobs.*
*Method 3: JEM intensity values applied only to jobs where participants reported the use of any RF-EMF occupational source.*

**Table S7.a.** Associations between categories of cumulative exposure to RF-EMF based on the 50th, 75th, and 90th percentiles and the risk of meningioma per exposure lag and time-window of exposure using < 50th percentile of cumulative exposure as the reference group. Electric fields (E).

| Exposure categories (ICNIRP squared ratio-years) | Exposure Assessment Method 1 | | | Exposure Assessment Method 2 | | | | Exposure Assessment Method 3 | | | |
|---|---|---|---|---|---|---|---|---|---|---|---|
| | Cases (n) | Controls (n) | OR [95% CI] | Exposure categories | Cases (n) | Controls (n) | OR [95% CI] | Exposure categories | Cases (n) | Controls (n) | OR [95% CI] |
| **1 year-lag** | | | | | | | | | | | |
| < 17.8 | 849 | 2470 | *1.00 (ref.)* | < 7.9 | 252 | 883 | *1.00 (ref.)* | < 3.6 | 61 | 328 | *1.00 (ref.)* |
| ≥ 17.8-40.2 | 410 | 1234 | 1.04 [0.81; 1.33] | ≥ 7.9-22.5 | 111 | 438 | 1.08 [0.81; 1.43] | ≥ 3.6-13.5 | 46 | 164 | 0.66 [0.49; 0.89] |
| ≥ 40.2-76.7 | 242 | 741 | 0.97 [0.75; 1.26] | ≥ 22.5-48.8 | 69 | 264 | 0.97 [0.69; 1.35] | ≥ 13.5-35.3 | 21 | 98 | 1.10 [0.77; 1.56] |
| ≥ 76.7 | 141 | 494 | 0.97 [0.74; 1.29] | ≥ 48.8 | 45 | 176 | 0.92 [0.62; 1.39] | ≥ 35.3 | 17 | 66 | 0.91 [0.55; 1.51] |
| **5 year-lag** | | | | | | | | | | | |
| < 15.2 | 833 | 2440 | *1.00 (ref.)* | < 7.5 | 235 | 851 | *1.00 (ref.)* | < 3.4 | 61 | 308 | *1.00 (ref.)* |
| ≥ 15.2-35.9 | 412 | 1219 | 1.02 [0.81; 1.28] | ≥ 7.5-21.3 | 110 | 428 | 1.12 [0.84; 1.49] | ≥ 3.4-12 | 39 | 154 | 0.69 [0.51; 0.93] |
| ≥ 35.9-68.8 | 232 | 732 | 0.95 [0.75; 1.22] | ≥ 21.3-43.9 | 62 | 253 | 0.93 [0.65; 1.31] | ≥ 12-33.3 | 18 | 92 | 0.97 [0.67; 1.42] |
| ≥ 68.8 | 146 | 488 | 0.93 [0.72; 1.22] | ≥ 43.9 | 44 | 170 | 0.92 [0.61; 1.39] | ≥ 33.3 | 18 | 62 | 0.83 [0.49; 1.43] |
| **10 year-lag** | | | | | | | | | | | |
| < 12.2 | 810 | 2362 | *1.00 (ref.)* | < 6.4 | 218 | 786 | *1.00 (ref.)* | < 2.9 | 50 | 275 | *1.00 (ref.)* |
| ≥ 12.2-30.2 | 401 | 1180 | 0.99 [0.81; 1.21] | ≥ 6.4-19.2 | 103 | 394 | 1.07 [0.80; 1.44] | ≥ 2.9-10.6 | 38 | 137 | 0.61 [0.44; 0.85] |
| ≥ 30.2-59.7 | 217 | 708 | 0.91 [0.73; 1.14] | ≥ 19.2-37.8 | 57 | 234 | 0.93 [0.64; 1.33] | ≥ 10.6-28 | 14 | 82 | 1.06 [0.72; 1.56] |
| ≥ 59.7 | 143 | 473 | 0.84 [0.66; 1.08] | ≥ 37.8 | 40 | 158 | 0.84 [0.55; 1.30] | ≥ 28 | 16 | 55 | 0.74 [0.41; 1.35] |
| **1 to 4 year time window** | | | | | | | | | | | |
| < 1.9 | 478 | 1753 | *1.00 (ref.)* | < 2.1 | 83 | 355 | *1.00 (ref.)* | < 1.4 | 22 | 134 | *1.00 (ref.)* |
| ≥ 1.9-4.8 | 284 | 890 | 0.89 [0.76; 1.03] | ≥ 2.1-4.7 | 43 | 174 | 1.37 [0.82; 2.28] | ≥ 1.4-3.8 | 10 | 66 | 0.72 [0.44; 1.15] |
| ≥ 4.8-9.2 | 179 | 531 | 0.94 [0.78; 1.12] | ≥ 4.7-11.8 | 39 | 109 | 1.68 [0.97; 2.92] | ≥ 3.8-9.4 | 14 | 40 | 0.71 [0.35; 1.43] |
| ≥ 9.2 | 93 | 320 | 0.99 [0.81; 1.23] | ≥ 11.8 | 13 | 67 | 0.61 [0.28; 1.34] | ≥ 9.4 | 8 | 27 | 1.42 [0.74; 2.73] |
| **5 to 9 year time window** | | | | | | | | | | | |
| < 2.9 | 566 | 1922 | *1.00 (ref.)* | < 3.1 | 119 | 425 | *1.00 (ref.)* | < 1.8 | 26 | 155 | *1.00 (ref.)* |
| ≥ 2.9-6.8 | 353 | 1054 | 0.94 [0.81; 1.10] | ≥ 3.1-6.4 | 46 | 219 | 0.81 [0.52; 1.27] | ≥ 1.8-5.8 | 17 | 85 | 0.71 [0.46; 1.10] |
| ≥ 6.8-12.8 | 148 | 469 | 0.95 [0.80; 1.13] | ≥ 6.4-15.3 | 24 | 120 | 0.69 [0.39; 1.23] | ≥ 5.8-14.6 | 12 | 44 | 0.88 [0.50; 1.52] |
| ≥ 12.8 | 106 | 383 | 1.01 [0.81; 1.27] | ≥ 15.3 | 22 | 83 | 0.81 [0.44; 1.50] | ≥ 14.6 | 12 | 26 | 1.06 [0.54; 2.09] |

*Conditional logistic regression models stratified by sex, age (5 year groups), countries and regions and adjusted for education.*
*Method 1: JEM intensity values applied to all exposed jobs.*
*Method 2: ISCO88 jobs having a prevalence of exposure falling below the median of JEM prevalence values (E fields: 5.1%; H fields: 4.8% for RF and 1.5% for IF-RF E and H fields) considered non-exposed; JEM intensity values applied to remaining jobs.*
*Method 3: JEM intensity values applied only to jobs where participants reported the use of any RF-EMF occupational source.*

**Table S7.b.** Associations between categories of cumulative exposure to RF-EMF based on the 50th, 75th, and 90th percentiles and the risk of meningioma per exposure lag and time-window of exposure using < 50th percentile of cumulative exposure as the reference group. Magnetic fields (H).

| Exposure categories (ICNIRP squared ratio-years) | Exposure Assessment Method 1 | | | Exposure Assessment Method 2 | | | | Exposure Assessment Method 3 | | | |
|---|---|---|---|---|---|---|---|---|---|---|---|
| | Cases (n) | Controls (n) | OR [95% CI] | Exposure categories | Cases (n) | Controls (n) | OR [95% CI] | Exposure categories | Cases (n) | Controls (n) | OR [95% CI] |
| **1 year-lag** | | | | | | | | | | | |
| < 2.1 | 858 | 2458 | *1.00 (ref.)* | < 1.1 | 270 | 858 | *1.00 (ref.)* | < 0.6 | 58 | 323 | *1.00 (ref.)* |
| ≥ 2.1-5.5 | 411 | 1228 | 1.03 [0.81; 1.31] | ≥ 1.1-3.2 | 100 | 429 | 0.8 [0.61; 1.06] | ≥ 0.6-1.8 | 40 | 162 | 0.63 [0.47; 0.86] |
| ≥ 5.5-17.9 | 213 | 737 | 0.99 [0.77; 1.27] | ≥ 3.2-10.2 | 56 | 259 | 0.81 [0.57; 1.15] | ≥ 1.8-6.5 | 23 | 95 | 0.91 [0.63; 1.32] |
| ≥ 17.9 | 152 | 492 | 0.91 [0.69; 1.20] | ≥ 10.2 | 42 | 170 | 0.79 [0.53; 1.20] | ≥ 6.5 | 21 | 65 | 1.05 [0.64; 1.70] |
| **5 year-lag** | | | | | | | | | | | |
| < 1.8 | 854 | 2425 | *1.00 (ref.)* | < 1 | 252 | 831 | *1.00 (ref.)* | < 0.5 | 56 | 305 | *1.00 (ref.)* |
| ≥ 1.8-4.9 | 410 | 1212 | 1.02 [0.82; 1.27] | ≥ 1-3.1 | 100 | 415 | 0.78 [0.58; 1.04] | ≥ 0.5-1.8 | 35 | 150 | 0.64 [0.47; 0.87] |
| ≥ 4.9-16.3 | 202 | 728 | 0.98 [0.77; 1.24] | ≥ 3.1-9.8 | 50 | 249 | 0.74 [0.51; 1.08] | ≥ 1.8-5.7 | 20 | 91 | 0.87 [0.59; 1.29] |
| ≥ 16.3 | 147 | 485 | 0.83 [0.64; 1.08] | ≥ 9.8 | 41 | 167 | 0.80 [0.52; 1.21] | ≥ 5.7 | 21 | 61 | 0.93 [0.56; 1.55] |
| **10 year-lag** | | | | | | | | | | | |
| < 1.4 | 834 | 2344 | *1.00 (ref.)* | < 0.9 | 234 | 777 | *1.00 (ref.)* | < 0.5 | 47 | 272 | *1.00 (ref.)* |
| ≥ 1.4-4.2 | 391 | 1171 | 1.01 [0.83; 1.23] | ≥ 0.9-2.9 | 93 | 388 | 0.81 [0.60; 1.08] | ≥ 0.5-1.6 | 33 | 135 | 0.58 [0.41; 0.81] |
| ≥ 4.2-14.5 | 194 | 703 | 0.91 [0.73; 1.13] | ≥ 2.9-8.9 | 44 | 233 | 0.71 [0.48; 1.05] | ≥ 1.6-5.1 | 16 | 82 | 0.93 [0.62; 1.41] |
| ≥ 14.5 | 141 | 469 | 0.80 [0.62; 1.03] | ≥ 8.9 | 40 | 156 | 0.88 [0.57; 1.35] | ≥ 5.1 | 18 | 54 | 0.76 [0.43; 1.33] |
| **1 to 4 year time window** | | | | | | | | | | | |
| < 0.2 | 465 | 1742 | *1.00 (ref.)* | < 0.2 | 87 | 327 | *1.00 (ref.)* | < 0.2 | 15 | 131 | *1.00 (ref.)* |
| ≥ 0.2-0.5 | 303 | 846 | 0.85 [0.73; 0.99] | ≥ 0.2-0.7 | 42 | 171 | 1.08 [0.66; 1.77] | ≥ 0.2-0.5 | 18 | 65 | 0.48 [0.27; 0.83] |
| ≥ 0.5-2 | 149 | 533 | 1.04 [0.88; 1.24] | ≥ 0.7-1.9 | 24 | 92 | 1.36 [0.72; 2.57] | ≥ 0.5-1.4 | 13 | 39 | 1.34 [0.76; 2.36] |
| ≥ 2 | 105 | 330 | 0.83 [0.67; 1.04] | ≥ 1.9 | 16 | 63 | 0.94 [0.45; 1.96] | ≥ 1.4 | 8 | 26 | 1.42 [0.73; 2.77] |
| **5 to 9 year time window** | | | | | | | | | | | |
| < 0.3 | 570 | 1899 | *1.00 (ref.)* | < 0.3 | 110 | 400 | *1.00 (ref.)* | < 0.3 | 23 | 152 | *1.00 (ref.)* |
| ≥ 0.3-0.7 | 349 | 1045 | 0.97 [0.83; 1.13] | ≥ 0.3-0.9 | 38 | 202 | 0.75 [0.47; 1.20] | ≥ 0.3-0.6 | 17 | 78 | 0.62 [0.39; 0.99] |
| ≥ 0.7-2.6 | 130 | 471 | 0.93 [0.79; 1.1] | ≥ 0.9-2.4 | 32 | 115 | 1.03 [0.60; 1.76] | ≥ 0.6-1.9 | 12 | 43 | 0.99 [0.57; 1.74] |
| ≥ 2.6 | 108 | 357 | 0.94 [0.74; 1.19] | ≥ 2.4 | 18 | 80 | 0.75 [0.38; 1.47] | ≥ 1.9 | 14 | 31 | 1.27 [0.64; 2.50] |

*Conditional logistic regression models stratified by sex, age (5 year groups), countries and regions and adjusted for education.*
*Method 1: JEM intensity values applied to all exposed jobs.*
*Method 2: ISCO88 jobs having a prevalence of exposure falling below the median of JEM prevalence values (E fields: 5.1%; H fields: 4.8% for RF and 1.5% for IF-RF E and H fields) considered non-exposed; JEM intensity values applied to remaining jobs.*
*Method 3: JEM intensity values applied only to jobs where participants reported the use of any RF-EMF occupational source.*

**Table S8.** Description of exposure per sex and ISCO88 1-digit occupation across the three methods of linking the INTEROCC RF-JEM to the occupational history of INTEROCC participants for E fields.

| Occupation (ISCO88 one digit group - Label) | n jobs (%) | Sex ratio (M:F) | % participants self-reporting use of specific RF-EMF source | | Exposure prevalence | | Exposure levels (ICNIRP ratios[2]) | |
|---|---|---|---|---|---|---|---|---|
| | | | Males | Females | Median | Max | Median | Max |
| Method 1 – All exposed jobs retained in the analysis | | | | | | | | |
| 1 – Managers | 2812 (8.3%) | 1924M:888F | 4.7 | 2.7 | 3.8 | 100.0 | 1.2 | 7.5 |
| 2 – Professionals | 5728 (16.9%) | 2622M:3106F | 3.5 | 2.0 | 1.0 | 33.3 | 0.1 | 10.1 |
| 3 - Technicians and associate professionals | 4511 (13.3%) | 2070M:2441F | 8.6 | 3.3 | 2.0 | 100.0 | 0.4 | 20.7 |
| 4 - Clerical support workers | 6427 (19%) | 1399M:5028F | 2.5 | 1.3 | 1.2 | 28.6 | 1.3 | 30.2 |
| 5 - Service and sales workers | 4047 (12%) | 1159M:2888F | 14.9 | 3.6 | 1.4 | 61.9 | 1.7 | 8.2 |
| 6 - Skilled agricultural, forestry and fishery workers | 480 (1.4%) | 342M:138F | 6.4 | 3.6 | 2.9 | 42.9 | 0.2 | 2.7 |
| 7 - Craft and related trades workers | 4063 (12%) | 3512M:551F | 7.3 | 2.0 | 3.8 | 100.0 | 0.9 | 28.3 |
| 8 - Plant and machine operators, and assemblers | 2446 (7.2%) | 1610M:836F | 10.1 | 3.5 | 4.8 | 40.0 | 0.4 | 34.0 |
| 9 - Elementary occupations | 2308 (6.8%) | 996M:1312F | 4.0 | 2.9 | 3.0 | 25.0 | 0.7 | 10.7 |
| A - Non-standard occupations | 1007 (3%) | 644M:363F | 18.6 | 1.1 | 12.1 | 12.1 | 0.2 | 0.5 |
| Method 2 - Only jobs having a prevalence of exposure ≥ median prevalence of exposure across all jobs considered as exposed | | | | | | | | |
| 1 - Managers | 1053 (3.1%) | 758M:295F | 8.0 | 6.4 | 7.1 | 100.0 | 1.6 | 7.5 |
| 2 - Professionals | 485 (1.4%) | 407M:78F | 9.3 | 2.6 | 7.7 | 33.3 | 0.9 | 10.1 |
| 3 - Technicians and associate professionals | 909 (2.7%) | 641M:268F | 20.4 | 16.4 | 14.3 | 100.0 | 0.4 | 9.0 |
| 4 - Clerical support workers | 166 (0.5%) | 68M:98F | 13.2 | 7.1 | 10.5 | 28.6 | 4.4 | 4.4 |
| 5 - Service and sales workers | 744 (2.2%) | 466M:278F | 29.4 | 20.5 | 21.8 | 61.9 | 0.0 | 3.9 |
| 6 - Skilled agricultural, forestry and fishery workers | 90 (0.3%) | 68M:22F | 16.2 | 13.6 | 10.0 | 42.9 | 0.4 | 2.7 |
| 7 - Craft and related trades workers | 1526 (4.5%) | 1499M:27F | 11.0 | 11.1 | 7.1 | 100.0 | 0.9 | 6.9 |
| 8 - Plant and machine operators, and assemblers | 725 (2.1%) | 642M:83F | 14.3 | 2.4 | 6.3 | 40.0 | 0.5 | 34.0 |
| 9 - Elementary occupations | 221 (0.7%) | 173M:48F | 11.0 | 8.3 | 8.0 | 25.0 | 3.8 | 10.7 |
| A - Non-standard occupations | 499 (1.5%) | 458M:41F | 25.8 | 9.8 | 12.1 | 12.1 | 0.5 | 0.5 |
| Method 3 - Only jobs where participants reported the use of a specific occupational RF-EMF source considered as exposed | | | | | | | | |
| 1 - Managers | 114 (0.3%) | 90M:24F | 100 | 100 | 5.2 | 100.0 | 1.2 | 7.5 |
| 2 - Professionals | 154 (0.5%) | 92M:62F | 100 | 100 | 1.8 | 30.8 | 0.1 | 10.1 |
| 3 - Technicians and associate professionals | 257 (0.8%) | 177M:80F | 100 | 100 | 20.4 | 100.0 | 0.4 | 20.7 |
| 4 - Clerical support workers | 98 (0.3%) | 35M:63F | 100 | 100 | 1.9 | 28.6 | 1.3 | 4.4 |
| 5 - Service and sales workers | 277 (0.8%) | 173M:104F | 100 | 100 | 21.8 | 61.9 | 0.0 | 8.2 |
| 6 - Skilled agricultural, forestry and fishery workers | 27 (0.1%) | 22M:5F | 100 | 100 | 6.0 | 42.9 | 0.2 | 2.7 |
| 7 - Craft and related trades workers | 266 (0.8%) | 255M:11F | 100 | 100 | 5.7 | 50.0 | 0.9 | 28.3 |
| 8 - Plant and machine operators, and assemblers | 192 (0.6%) | 163M:29F | 100 | 100 | 6.3 | 40.0 | 0.5 | 28.3 |
| 9 - Elementary occupations | 78 (0.2%) | 40M:38F | 100 | 100 | 3.0 | 25.0 | 1.4 | 10.7 |
| A - Non-standard occupations | 124 (0.4%) | 120M:4F | 100 | 100 | 12.1 | 12.1 | 0.5 | 0.5 |
